# Evaluating Reproductive Health Recommendations in CPGs for Sickle Cell Disease: An Umbrella Review

**DOI:** 10.1002/gin2.70091

**Published:** 2026-09-22

**Authors:** Lisa R. Roberts, Marc Julmisse, Safiye Sahin, Jan M. Nick, Shanalee Tamares

**Affiliations:** ^1^ Loma Linda University School of Nursing Loma Linda California USA; ^2^ JBI Center for Evidence Synthesis Loma Linda University Health Loma Linda California USA; ^3^ Loma Linda University Libraries Loma Linda California USA

**Keywords:** clinical practice guidelines, PICAR framework, reproductive health recommendations, sickle cell disease, umbrella review

## Abstract

**Background:**

Individuals with sickle cell disease or trait (SCD/T) face significant reproductive health risks, highlighting the need to assess the best available evidence on their reproductive health needs.

**Objectives:**

To assess the quality of SCD/T clinical practice guidelines and evaluate the key characteristics and quality of their reproductive health recommendations.

**Methods:**

This systematic review followed the JBI methodology for Umbrella Reviews. Eligible CPGs required RHRs for individuals with SCD/T. Databases included PubMed, CINAHL, PsycINFO, Cochrane, Embase, Web of Science, Google Scholar, and registries, for CPGs in March 2025 without language restriction. Screening followed a three‐step process with two independent reviewers. Risk of bias was assessed using AGREE‐II and AGREE‐Rex. Data was extracted independently by two reviewers using a tool developed by the researchers. Conflicts between independent reviewers were resolved through discussion. Findings were synthesized using descriptive analyses, frequency tables, and narrative summaries. One deviation from the protocol is documented in the final review.

**Results:**

Fifteen CPGs published between 2020 and 2025 were included, most of which were national‐level and peer‐reviewed. AGREE II scores ranged from 48% to 97%, with Applicability scoring lowest and six CPGs recommended for use without modification. Across the guidelines, 198 reproductive health recommendations were identified. Most recommendations focused on antenatal, intrapartum, and postnatal care (64.7%) and management‐related care stages (62.1%). Safe abortion care appeared in only one guideline. Although nine CPGs used GRADE, many did not clearly report recommendation strength or evidence certainty. AGREE‐REX identified seven CPGs as suitable for use without modification, with Values and Preferences scoring lowest. Inter‐rater reliability was high for both tools.

**Conclusions:**

These findings highlight the need for more comprehensive, evidence‐based, and patient‐centered guidelines and recommendations to support high‐quality reproductive healthcare for individuals with SCD.

**Umbrella Review Registration Number:**

The protocol was registered in PROSPERO, CRD42025648554.

## Summary

**What We Already Know:**

- Individuals with sickle cell disease face substantial reproductive health risks throughout the life course, while sickle cell trait has important implications for carrier screening, partner testing, genetic counseling, and reproductive planning.
- Reproductive health recommendations are distributed across multiple clinical practice guidelines, and their key characteristics and quality have not been comprehensively evaluated and synthesized.

**What This Adds to What Was Known:**

- This umbrella review evaluated 15 clinical practice guidelines published between 2020 and 2025 and identified 198 reproductive health recommendations.
- Guideline quality varied considerably, with overall AGREE II scores ranging from 48% to 97%. Among the assessed domains, Applicability received the lowest AGREE II score, while Values and Preferences received the lowest AGREE‐REX score.
- Most recommendations focused on antenatal, intrapartum, and postnatal care, while major gaps remained in safe abortion care. Recommendations concerning fertility, contraception, and genetic counseling were also limited or inconsistently reported.

**What Is the Implication and What Should Change Now?:**

- Guideline developers should broaden reproductive health guidance beyond pregnancy‐related care and provide more comprehensive recommendations on safe abortion care, fertility, contraception, genetic counseling, and preconception care.
- Future guidelines should improve applicability, incorporate patients' values and preferences, and transparently report recommendation strength and certainty of evidence.
- This synthesis can help healthcare providers identify existing recommendations and recognize areas in which further evidence and guideline development are needed.

**Abbreviations**
AGREE‐II: Appraisal of Guidelines for Research and Evaluation II
AGREE‐REX: Appraisal of Guidelines Research and Evaluation–Recommendations Excellence
AI: artificial intelligence
CIs: confidence intervals
CPGs: clinical practice guidelines
GRADE: grading of recommendations assessment, development, and evaluation
HICs: high‐income countries
IBM SPSS: International Business Machines Statistical Package for the Social Sciences
ICC: intraclass correlation coefficient
IGO: intergovernmental organization
JBI SUMARI: JBI system for the unified management, assessment, and review of information
LMICs: low‐ and middle‐income countries
LOTE: languages other than English
MeSH: medical subject headings
NGO: non‐governmental organization
Oxford: Oxford center for evidence‐based medicine
PICAR: population, intervention, comparators, attributes of recommendations
PPI: patient and public involvement
PRESS: peer review of electronic search strategies
PRISMA: Preferred Reporting Items for Systematic Reviews and Meta‐Analyses
RHRs: reproductive health recommendations
SCD: sickle cell disease
SCD/T: sickle cell disease/trait
SCT: sickle cell trait
SIGN: Scottish intercollegiate guidelines network
SPSS: Statistical Package for the Social Sciences
USA: United States of America
VOCs: vaso‐occlusive crisis
WHO: World Health Organization

## 1 Introduction

Globally, sickle cell disease (SCD) is the most prevalent inherited blood disorder, affecting approximately 8 million worldwide and about 100,000 individuals in the United States [1, 2]. A genetic mutation alters the structure and function of red blood cells causing vaso‐occlusive crises (VOCs), a hallmark of SCD. VOCs result in not only acute and chronic pain, but end‐organ damage, reduced quality of life, and shortened lifespan [2]. Global estimates of SCD prevalence and burden are limited by under‐recognition of its impact on all‐cause mortality, under‐5 mortality, premature death, and unmeasured morbidity. SCD and sickle cell trait (SCT) disproportionately affect individuals of ancestry from malaria‐endemic regions, such as parts of Africa, Asia, the Middle East, and the Hispanic diaspora [2].

More than 300 million people worldwide are estimated to have SCT [3, 4]. Although SCT is generally asymptomatic and does not present the same clinical reproductive health concerns as SCD, its reproductive implications are important [4, 5]. Because individuals may be unaware of their carrier status, the sickle cell gene can be unknowingly transmitted to future generations [5]. Carrier screening, partner testing, and genetic counseling within primary and preconception care can identify reproductive risks and support informed decision‐making [5, 6]. These potentially cost‐effective services may have a substantial impact by helping individuals understand the likelihood of having a child with SCD [6]. Therefore, this review includes reproductive health needs of individuals with SCT.

With advances in treatment, survival has improved, and many people with SCD are now living into their reproductive years [7]. Reproductive health concerns are highly relevant among individuals with SCD, affecting men (e.g., hypogonadism, low testosterone, erectile dysfunction, infertility, and sperm abnormalities) [8, 9] and women (delayed menarche, heavy menstrual bleeding, increased frequency of VOCs with menstruation, sexual dysfunction, diminished ovarian reserve, and infertility) [9, 10]. Furthermore, contraceptive decision‐making is challenged by the hypercoagulability associated with SCD, which narrows the range of safe pregnancy‐prevention options. Comprehensive reproductive counseling is therefore essential for all individuals with SCD, regardless of their childbearing intentions.

Maternal mortality among women with SCD was 26 times higher than non‐Black women without SCD, and 10 times higher than Black women without SCD in a recent national study of birthing women in the U.S. [11]. Women with SCD, and to a lesser extent those with SCT, are also at risk for maternal morbidity [11, 12]. The frequency and intensity of sickle crises in women with SCD is increased by 35%–50% during pregnancy [13]. The risks for preeclampsia, stillbirth and preterm delivery, and giving birth to small‐for‐gestational‐age infants and other untoward outcomes are also increased [14]. Given these risks, CPGs that address reproductive health for individuals with SCD are essential. However, current guidance remains fragmented, and a recent scoping review found that recommendations on fertility, family building, and contraception for adolescents and young adults with SCD are limited and inconsistently applied [15]. Furthermore, variable methodological rigor across CPGs can leave clinicians, patients, and policymakers unsure which recommendations are most reliable, leading to inconsistent care and heightened risk of adverse reproductive outcomes among this vulnerable population.

Although umbrella reviews are traditionally defined as reviews of systematic reviews, in many clinical fields the most robust synthesized evidence takes the form of clinical practice guidelines. Because our objective was to synthesize recommendations rather than intervention effects, we applied a synthesis science approach to conduct a review of CPGs. This approach aligns with the methodological intent of umbrella reviews while reflecting the available evidence base. Such a review enabled systematic assessment of methodological rigor and recommendation quality, providing a comprehensive synthesis to inform clinical decision‐making and policy development in reproductive health for individuals with SCD.

Specifically, the objectives of this umbrella review were to (1) identify the methodological rigor of CPGs on SCD that include reproductive health recommendations, and (2) synthesize the content and quality of those recommendations. A comprehensive search of PROSPERO, MEDLINE, the Cochrane Database of Systematic Reviews, and JBI Evidence Synthesis confirmed that no current or ongoing umbrella reviews exist on this topic. Furthermore, an initial probing PubMed search identified 360 CPG records for SCD, which confirmed that sufficient data existed to support a robust synthesis.

## 2 Description of Review

This review employed the PICAR framework for articulating the review question when doing a systematic review of clinical practice guidelines, as suggested by Johnston et al. [16]: **P**opulation, clinical indication(s), and condition(s); **I**ntervention(s) or **I**nterest area; **C**omparator(s)/Comparisons(s), or (key) Content; **A**ttributes of eligible CPGs; and **R**ecommendation characteristics.

Review questions:

1. What are the attributes and methodological rigor of clinical practice guidelines that include reproductive health recommendations (RHRs) for individuals with sickle cell disease?
2. What are the characteristics, including care domains, stages of the care continuum, and grading system, as well as quality of individual RHRs found within SCD clinical practice guidelines?

### 2.1 Inclusion Criteria

#### 2.1.1 Participants

This umbrella review included CPGs that focused on the management of individuals with SCD from all ethnicities and any gender, during their reproductive years. We used the World Health Organization (WHO) defined reproductive age range for females of 15–49 years [17]. The reproductive age for males was defined as starting between 9 and 14 years of age, with no specific upper age limit for reproductive potential [18].

#### 2.1.2 Interest Area

The area of interest was CPGs that included RHRs applicable in any setting (e.g., acute care, community, and outpatient clinic) for managing individuals with SCD. Guidelines without RHRs were excluded. CPGs with either the term “guideline” or the condition “Sickle cell” in the title were considered; the term recommendation/s, however, did not have to be in the title. RHRs were considered across the preconception to postnatal continuum, and included fertility care, safe abortion care, and genetic counseling for individuals with SCD at any stage of care (screening, diagnosis, risk reduction, or management).

#### 2.1.3 Comparator

No comparators were included, rather, key content (per PICAR framework) was sought. Key content included RHRs for individuals with sickle cell disease.

#### 2.1.4 Attributes of Eligible CPGs

To be included in this umbrella review, CPGs had to originate from authoritative sources, such as governmental bodies, non‐governmental organizations, or professional organizations, at international, national, state, or local levels. National CPGs may have been developed within a single country, while international CPGs were typically issued by organizations operating across multiple countries. The review included CPGs addressing both pharmacological and non‐pharmacological interventions. Both evidence‐based and consensus‐based recommendations were considered, and efforts were made to capture guidelines from both high‐income countries (HICs) and low‐ and middle‐income countries (LMICs). Intended end‐users of the CPGs were clinicians, patients, and policymakers.

Exclusion criteria consisted of practice recommendations from a non‐authoritative source or without organizational endorsement. Additionally, CPGs were excluded if (1) they were outdated, were retracted, or lacked clinical focus; (2) the primary focus was on the management of other medical conditions or provided “general management considerations”; (3) CPGs were developed by individual institutions; (4) CPGs did not contain any reproductive health recommendations. As only CPGs were included in this umbrella review, primary, original, single studies, as well as text and opinion pieces were excluded.

### 2.2 Reproductive Health Recommendation (RHR) Characteristics

For inclusion in this review, recommendations had to specifically focus on reproductive health care. RHRs can be about (a) antenatal, intrapartum, and postnatal care, (b) contraception counseling and provision, (c) fertility care, (d) safe abortion care, and (e) genetic counseling [19]. All stages of the care continuum addressed by the recommendation (screening, diagnosis, risk reduction, or management) [20] were also included. The RHRs did not need to be explicitly highlighted but needed to be found or noted within the body of the document. To be eligible, recommendations could have included an explicit level of confidence (and specified quality assessment criteria system used), but this was not required. Recommendations falling outside the reproductive age range (i.e., pre‐pubescent males and females, menopausal, or post‐menopausal women) were excluded from consideration.

## 3 Methods

This systematic review was conducted in accordance with the JBI methodology for Umbrella reviews [21]. The protocol was registered in PROSPERO, CRD42025648554 and is under review. Deviations from the protocol are reported and justification is provided in the appropriate section of the review.

### 3.1 Search Strategy

The search strategy aimed to identify clinical practice guidelines worldwide, acknowledging the increasingly global distribution of SCD driven by migration and population mobility. This inclusive approach aimed to provide robust data to approximate the totality of empirical evidence available. A comprehensive search was achieved utilizing a three‐step search strategy. CPGs published in any major language were included in the search to reduce publication bias and increase global representation. Publications in languages other than English (LOTE) were to be translated to English using an AI Large‐language‐model, which includes more than 30 major languages, however, only CPGs in English were found. To ensure that the review reflected current clinical practice, only CPGs published from 2020 onward were included. This timeframe was selected to prioritize the most recent recommendations and reduce the inclusion of guidance that may no longer reflect current evidence or clinical practice.

First, an initial limited search of PubMed was conducted using MeSH terms and subject heading terms from the elements of the research questions. The text words found in titles and abstracts of relevant articles were used to develop search strings for databases searched. A pilot test of the developed search string, which utilized the Guideline search filter from Canada's Drug Agency Search Filters Database, can be seen in Additional File 1.

In the second step, the library scientist used the MeSH terms and indexed words identified in the first step and adapted the strategy for each specific database and/or information source. Databases used for the comprehensive search included: CINAHL and PsycINFO on the EBSCOhost platform, Cochrane Library, PubMed (using the validated filter for CPGs) [22], DynaMed, Embase (Platform), Trip Medical Database, Web of Science, and Google Scholar will be conducted. Registries searched included Guidelines International Network (GIN), National Guidelines Clearinghouse (NGC) archived, Epistemonikos, WHO Guidelines Repository, National Institute for Health and Care Excellence (NICE), CMA Infobase, and Guidelines Central. Gray literature searching included specialty organizational websites on SCD and using NGO Search (Google) and IGO Search strategies (Google), and any conference proceedings; however, only the first 200 sources were downloaded from Google Scholar per recommendations [23, 24]. The search strategy developed for each database (and platform) was peer‐reviewed by a library scientist prior to execution, following best practices, as per the PRESS checklist [25].

The third step consisted of forward and backward citation searching using Web of Science and Google Scholar, with supplementary hand searching of references from articles identified in step 2. Additional records found are reflected in the PRISMA 2020 flow diagram under “Identification of other studies via other methods.”

All results from the databases, trial registries, and gray literature were collated and placed into EndNote v. 21 (Clarivate Analytics, Pennsylvania, USA); duplicate citations were removed.

CPG Screening and SelectionCitation details of the located studies were imported into the JBI System for the Unified Management, Assessment, and Review of Information (JBI SUMARI; JBI, Adelaide, Australia) [26]. CPG selection was conducted following a three‐step screening process. First, after a pilot test of five citations, with at least 80% agreement, two reviewers independently screened the titles and abstracts of the retrieved guidelines to determine their relevance to the review objectives and study population.

In the second step, citations that passed the first step of screening were subject to a full‐text review by two reviewers who independently assessed alignment against the inclusion and exclusion criteria. The library scientist procured the full‐text reports and uploaded them to JBI SUMARI. Reasons for full‐text reports being excluded were recorded and are reported in Additional File 2.

The third step of screening and study selection involved assessment of CPG quality and appraisal of recommendation quality. Two reviewers independently conducted the critical appraisals to determine if reports being screened for merited inclusion. Disagreements that arose between the reviewers at any stage of the selection process were resolved through discussion. The results of the search are reported and presented in the PRISMA 2020 flow diagram [27], see Figure 1.

**Figure 1:**
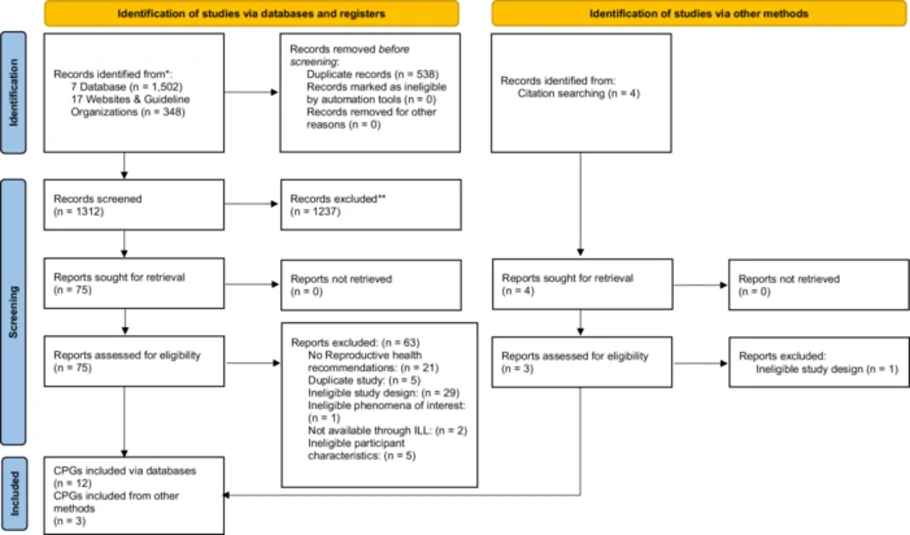
PRISMA 2020 flow diagram for the umbrella review which included searches of databases, registers, and other sources. *Total from all databases, and websites/organizations. **No automation tools were used. This work is licensed under CC BY 4.0. To view a copy of this license, visit https://creativecommons.org/licenses/by/4.0/ *Source:* Page MJ, et al. BMJ 2021;372:n71. doi: 10.1136/bmj.n71.

### 3.2 Assessment of CPG Rigor and RHR Quality

CPG quality was assessed using the Appraisal of Guidelines for Research and Evaluation II (AGREE II) instrument [28]. The AGREE II, a revised version of the original instrument, is composed of 23 items in six domains, which allowed developers to gauge the integration of patient benefits, side effects, risks, supporting evidence, feedback, and a plan for updates as new evidence emerges. To safeguard against bias, and ensure transparency, reproducibility, and contextual relevance, CPGs across low‐, middle‐, and high‐resource settings were sought. To achieve comprehensiveness, all CPGs on SCD that had RHRs were included, regardless of their quality assessment score.

The AGREE‐REX (Appraisal of Guidelines for Research and Evaluation Recommendation Excellence) instrument was used to assess the excellence of RHRs [29]. This psychometrically sound instrument consists of 9 items across three domains: clinical credibility, values and preferences, and implementability. Each item is rated on a 7‐point scale (1 = lowest quality, 7 = highest quality). Based on the item scores, the evaluation concludes with two summary statements regarding the recommendation's suitability for managing care in terms of content and context. Each statement is rated as “Yes,” “Yes with modifications,” or “No.” Threshold scores were determined by using: High quality, overall score > 70%; Moderate quality, score between 30% and 70%; Low quality, score < 30%. AGREE‐REX results were used to describe recommendation quality during the synthesis stage. The results of appraisals for CPG quality and RHR quality are reported in narrative and tabular formats.

### 3.3 Data Extraction

Two reviewers extracted data from the final selection of CPGs using a created tool for extraction of standardized information specifically for umbrella review of CPGs (see Additional File 3). The data extracted includes specific details to describe the demographic attributes of the CPG, such as the type of the publication (website or peer‐reviewed publication), year of publication, publishing organization (Government, professional organization, or international guideline organization), geographic location for the intended use, language of publication, population focus, and target audience of the CPG (e.g., clinicians, patients, and policymakers), number of recommendations in each CPG, as well as how many RHR were found in the CPG. Lastly, data extraction documented the tool used to determine the level of evidence quality and strength of the recommendation (GRADE, Oxford, SIGN, Shekelle Evidence Rating System, or not mentioned). The extraction of data for the characteristics of CPG rigor is documented using the results from the AGREE‐II instrument.

The extraction tool also characterizes RHR domains, purpose, and grading. We used the reproductive domains provided by the WHO [19] to create extraction categories: (a) antenatal, intrapartum, and postnatal care, (b) contraception counseling and provision, (c) fertility care, and (d) safe abortion care. A fifth category, added by the authors, includes genetic counseling. The team also extracted data on the stage of the care continuum addressed by the recommendation (screening, diagnosis, risk reduction, or management) [20]. We then extracted data on the RHR grading of evidence using strength of recommendation (strong or weak), level of evidence (High, Moderate, Low, and Very low), and the approach for determining the ratings (i.e., consensus, evidence‐based, and a priori specification) as available. We did not extract data at the level of individual recommendations. Instead, we extracted data collectively for all recommendations within each CPG, following one of the approaches outlined in the AGREE‐REX tool. Finally, the standardized AGREE‐REX instrument results were extracted to grade the quality of each recommendation.

Strategies used to minimize errors during data extraction included in‐person training to improve consistency and reliability, simultaneous review, and group review of extracted data to standardize entries. Disagreements between the reviewers were resolved through discussion. CPGs were evaluated as published, and no authoring organizations were contacted.

### 3.4 Data Synthesis

The above data extracted from included CPGs was tabulated and is accompanied by narrative synthesis to address the review objectives and the two specific questions. Descriptive measures were used to summarize the demographic attributes of the included CPGs. For methodological quality appraisal, AGREE II and AGREE‐REX domain scores and scaled percentages were calculated. Inter‐rater reliability between the two reviewers was assessed using SPSS (IBM SPSS Statistics v. 31), applying a two‐way random‐effects model with absolute agreement to calculate intraclass correlation coefficients (ICC). Descriptive statistics and 95% confidence intervals (CIs) for domain scores were also generated in SPSS for both AGREE II and AGREE‐REX results. Tables with domain scores and percentages are used to portray methodological rigor in the included CPGs, based on AGREE‐II results.

Additionally, Microsoft Excel pivot tables were used to analyze and summarize the characteristics of the included CPGs and reproductive health–related recommendations. Frequency data analytics tables were created to clearly present these categorical analyses. Tables and figures with frequency data analytics are used to portray the WHO domains, continuum of care, and the grading systems used for RHR. The quality of RHRs are displayed using tables that portray AGREE‐REX domain scores and percentages.

### 3.5 Patient and Public Involvement (PPI)

This umbrella review was developed in consultation with community‐based organization partners and patients with SCD. PPI stakeholders reviewed the protocol and gave input into the review objectives, conduct, and data extraction details.

## 4 Results

Fifteen CPGs met inclusion criteria by presenting RHRs for individuals with SCD [30, 31, 32, 33, 34, 35, 36, 37, 38, 39, 40, 41, 42, 43, 44].

### 4.1 Summary Attributes of the CPGs

As summarized in Table 1, the included CPGs represented a wide range of geographic locations. Included CPGs were developed for use at national (e.g., Australia, Italy, United Kingdom, and USA) [30, 31, 33, 34, 35, 36, 37, 39, 40, 41, 42, 43], regional (Europe) [38], institutional (single U.S. center) [41], and global (WHO) [44] levels. Publication dates ranged from 2020 to 2025, with nearly half (*n* = 7, 46.7%) [32, 34, 35, 37, 40, 41, 44] published in 2024 and 2025. Most CPGs (*n* = 12, 80%) were peer‐reviewed [30, 31, 32, 33, 36, 37, 38, 39, 40, 42, 43, 44]. The inclusion of a single institutional CPG [41] is a deviation from the protocol. The decision to include it was made due to the open‐access nature of the guideline. This accessibility could exert influence on clinical practice. Given its potential for adoption by other institutions or clinicians, evaluating its quality and methodological rigor was important to help inform users about the reliability of its recommendations.

**Table 1:** Summary attributes for clinical practice guidelines (CPGs).

Guideline	Intended location/country for use	Focus of CPG*	Target audience	Peer‐reviewed
American Society of Hematology 2020 guidelines for sickle cell disease: management of acute and chronic pain (2020) [28]	USA	Individuals with Sickle Cell Disease; children, adolescents, adults; includes pregnancy	Healthcare Providers Patients Policymakers Researchers	Peer‐reviewed Publication
American Society of Hematology 2020 guidelines for sickle cell disease: transfusion support (2020) [29]	USA	Individuals with Sickle Cell Disease (SCD; Children, adolescents, and adults; Includes pregnant individuals with SCD	Healthcare Providers Patients Policymakers Researchers	Peer‐reviewed Publication
Consensus of the Brazilian Association of Hematology, Hemotherapy and Cellular Therapy (ABHH) and the Brazilian Ministry of Health – General management of blood and blood products on the tests necessary for the release of exceptional medicines for sickle (2024) [30]	Brazil	Individuals with Sickle Cell Disease (SCD); Includes children, adolescents, and adults; Addresses women of childbearing age	Healthcare Providers Patients Policymakers	Peer‐reviewed Publication
First and Second Level Haemoglobinopathies Diagnosis: Best Practices of the Italian Society of Thalassemia and Haemoglobinopathies (SITE) (2022) [31]	Italy	Individuals with hemoglobinopathies, including sickle cell disease and thalassemia Pregnant women and at‐risk couples (prenatal diagnosis focus)	Healthcare Providers Patients	Peer‐reviewed Publication
Hemoglobinopathies in Pregnancy; Practice Advisory (2025) [American College of Obstetricians and Gynecologists] [32]	USA	Pregnant women with hemoglobinopathies	Healthcare Providers	Other: developed by experts
Management of acute presentations of sickle cell disease in the emergency department (2024) [The Royal College of Emergency Medicine] [33]	UK	Individuals with Sickle Cell Disease (SCD) Children, adolescents, and adults. Includes pregnant individuals with SCD presenting acutely	Healthcare Providers	National Professional Organizational Website
Management of sickle cell disease in pregnancy. A British Society for Hematology Guideline (2021) [34]	UK	Women with sickle cell disease (SCD)	Healthcare Providers	Peer‐reviewed Publication
Position statement on the management of pregnancy in sickle cell disease (2025) [The Australian Sickle Cell Disease Working Group] [35]	Australia	Patients of childbearing age in high‐risk groups, Women/patients with sickle cell disease (SCD)	Healthcare Providers	Peer‐reviewed Publication
Recommendations for Pregnancy in Rare Inherited Anemias (2020) [European Hematology Association] [36]	Europe	Women of reproductive age/pregnant individuals with rare inherited anemias	Healthcare Providers	Peer‐reviewed Publication
Significant haemoglobinopathies: a guideline for screening and diagnosis: a British Society for Hematology guideline (2023) [37]	UK	Patients of childbearing age in high‐risk groups	Healthcare Providers Patients Policymakers	Peer‐reviewed Publication
Society for Maternal‐Fetal Medicine Consult Series #68: Sickle cell disease in pregnancy (2024) [38]	USA	Pregnant and reproductive‐aged individuals with sickle cell disease	Healthcare Providers	Peer‐reviewed Publication
Standard of Care Guidelines for Patients with Sickle Cell Disease (2024) [UCSF Center of Excellence Staff] [39]	Single institution in USA	Pregnant and reproductive‐aged individuals with sickle cell disease	Healthcare Providers	Institutional internal guideline
Thalassemias and Sickle Cell Diseases in Pregnancy: SITE Good Practice (2022) [40]	Italy	Pregnant and reproductive‐aged individuals with sickle cell disease and thalassemia	Healthcare Providers	Peer‐reviewed Publication
The Diagnosis and Management of Priapism: AUA/SMSNA Guideline (2022) [41]	USA	Priapism in male patients with sickle cell disease	Healthcare Providers	Peer‐reviewed Publication
WHO recommendations on the management of sickle cell disease during pregnancy, childbirth and the interpregnancy period (2025) [42]	Global	Pregnant and reproductive‐aged individuals with sickle cell disease	Healthcare Providers Policymakers Professional societies	Peer‐reviewed Publication

*Notes:* LoE, level of evidence tool used to grade the quality and certainty of evidence; RHR, reproductive health recommendations.

* As reported by the authors.

All guidelines identified healthcare providers as their primary target audience and intended users. One‐third also targeted patients and policy makers (33% each) [30, 31, 33, 39, 44], while only two (13.34%) targeted researchers [30, 31]. The population focus for all included CPGs was individuals with SCD. However, specific characteristics such as gender and age range varied.

### 4.2 Methodological Rigor of CPGs

Methodological rigor, assessed using AGREE II, varied across guidelines. Overall Assessment scores ranged from 48% to 97%. Six CPGs were deemed appropriate to recommend for use without modification [30, 31, 38, 42, 43, 44], while nine were assessed as requiring modification [32, 33, 34, 35, 36, 37, 39, 40, 41]. Across the six AGREE II domains, Applicability (Domain 5) received the lowest mean score (61%). In addition to low scores in Applicability, mean scores for Rigor of Development (62%) and Editorial Independence (66%) were modest, with Editorial Independence demonstrating the most variability across guidelines, whereas Scope and Purpose (Domain 1) received the highest (89%). Inter‐rater reliability for AGREE II scoring was high across domains (ICCs 0.75–0.96), indicating strong agreement between researchers. Detailed scores are presented in Table 2.

**Table 2:** AGREE II domain scores and recommendations for use.

Guideline	Domain 1 scope & purpose	Domain 2 stakeholder involvement	Domain 3 rigor of development	Domain 4 clarity of presentation	Domain 5 applicability	Domain 6 editorial independence	Overall assessment	Recommend for use
American Society of Hematology 2020 guidelines for sickle cell disease: management of acute and chronic pain (2020) [28]	100%	100%	97%	100%	85%	100%	97%	R
American Society of Hematology 2020 guidelines for sickle cell disease: transfusion support (2020) [29]	100%	100%	97%	100%	85%	100%	97%	R
Consensus of the Brazilian Association of Hematology, Hemotherapy and Cellular Therapy (ABHH) and the Brazilian Ministry of Health – General management of blood and blood products on the tests necessary for the release of exceptional medicines for sickle (2024) [30]	94%	47%	25%	69%	46%	17%	50%	RM
First and Second Level Haemoglobinopathies Diagnosis: Best Practices of the Italian Society of Thalassemia and Haemoglobinopathies (SITE) (2022) [31]	75%	56%	63%	78%	77%	75%	70%	RM
Hemoglobinopathies in Pregnancy; Practice Advisory (2025) [American College of Obstetricians and Gynecologists] [32]	69%	47%	33%	69%	52%	17%	48%	RM
Management of acute presentations of sickle cell disease in the emergency department (2024) [The Royal College of Emergency Medicine] [33]	83%	75%	36%	75%	58%	42%	62%	RM
Management of sickle cell disease in pregnancy. A British Society for Hematology Guideline (2021) [34]	100%	53%	74%	100%	27%	83%	73%	RM
Position statement on the management of pregnancy in sickle cell disease (2025) [The Australian Sickle Cell Disease Working Group] [35]	86%	61%	32%	72%	50%	79%	63%	RM
Recommendations for Pregnancy in Rare Inherited Anemias (2020) [European Hematology Association] [36]	97%	78%	76%	69%	69%	96%	81%	R
Significant haemoglobinopathies: a guideline for screening and diagnosis: a British Society for Hematology guideline (2023) [37]	58%	58%	64%	75%	48%	58%	60%	RM
Society for Maternal‐Fetal Medicine Consult Series #68: Sickle cell disease in pregnancy (2024) [38]	97%	64%	51%	89%	48%	75%	71%	RM
Standard of Care Guidelines for Patients with Sickle Cell Disease (2024) [UCSF Center of Excellence Staff] [39]	89%	89%	39%	78%	69%	4%	61%	RM
Thalassemias and Sickle Cell Diseases in Pregnancy: SITE Good Practice (2022) [40]	94%	81%	76%	72%	75%	88%	81%	R
The Diagnosis and Management of Priapism: AUA/SMSNA Guideline (2022) [41]	100%	64%	86%	100%	44%	67%	77%	R
WHO recommendations on the management of sickle cell disease during pregnancy, childbirth, and the interpregnancy period (2025) [42]	97%	83%	88%	94%	83%	100%	91%	R
Domain mean	89%	70%	62%	83%	61%	66%	72%	
Standard deviation	13%	17%	24%	13%	18%	32%	15%	
95% Confidence Intervals (CIs) for Mean	82%–96%	61%–80%	44%–70%	76%–90%	45%–65%	49%–84%	64%–80%	
Intraclass Correlation Coefficient (ICC) (Rater 1 and 2)	0.96	0.84	0.95	0.75	0.76	0.90	0.90	

*Notes:* Overall assessment was calculated by averaging all individual items included in the tool.

R, recommended for use; RM, recommended for use with modifications; NR, not recommended for use.

In this dataset, the SDs (ranging from 0.13 to 0.32) are all below 0.35, which suggests moderate spread.

ICCs: calculated absolute ICCs using SPSS v. 32.

### 4.3 Summary Attributes of Reproductive Health Recommendations

Across the 15 included CPGs, a total of 198 RHRs were identified. The total number of recommendations within each CPG ranged from 3 [34] to 62 [41]. The number of RHRs per guideline varied substantially, ranging from 1 [31] to 60 [36] with an average of 13.2 RHRs per CPG. The summary attributes for RHRs can be seen in Table 3.

**Table 3:** Summary attributes for reproductive health recommendations.

Guideline	# of any recommendations in CPG*	# of RHRs** in CPG*	Level of evidence (LoE) tool used	Strength of the RHRs** in the CPG*	Level/quality of the body of evidence for RHR**	WHO care domains of RHR**	Stage of care continuum of RHR**
American Society of Hematology 2020 guidelines for sickle cell disease: management of acute and chronic pain (2020) [28]	10	2	GRADE	Weak	Low; Very Low	Antenatal, Intrapartum, and Postnatal Care	Risk reduction
American Society of Hematology 2020 guidelines for sickle cell disease: transfusion support (2020) [29]	10	1	GRADE	Weak	Very Low	Antenatal, Intrapartum, and Postnatal Care Fertility Care	Risk reduction
Consensus of the Brazilian Association of Hematology, Hemotherapy and Cellular Therapy (ABHH) and the Brazilian Ministry of Health – General management of blood and blood products on the tests necessary for the release of exceptional medicines for sickle (2024) [30]	4	2	Not mentioned	Not specified	Not specified	Antenatal, Intrapartum, and Postnatal Care	Screening Risk reduction
First and Second Level Haemoglobinopathies Diagnosis: Best Practices of the Italian Society of Thalassemia and Haemoglobinopathies (SITE) (2022) [31]	25	13	GRADE	Not specified	Not specified	Contraception Counseling and Provision Genetic Counseling	Screening Diagnosis Risk reduction Management
Hemoglobinopathies in Pregnancy; Practice Advisory (2025) [American College of Obstetricians and Gynecologists] [32]	3	3	Not mentioned	Not specified	Not specified	Antenatal, Intrapartum, and Postnatal Care Genetic Counseling	Screening Management
Management of acute presentations of sickle cell disease in the emergency department (2024)	10	5	Not mentioned	Not specified	Not specified	Antenatal, Intrapartum, and Postnatal Care Contraception Counseling and Provision Fertility Care	Screening Risk reduction Management
[The Royal College of Emergency Medicine] [33]	60	60	GRADE	Strong; Weak	High; Moderate; Low; Very Low	Antenatal, Intrapartum, and Postnatal Care Contraception Counseling and Provision Fertility Care Safe abortion Care Genetic Counseling	Screening Diagnosis Risk reduction Management
Management of sickle cell disease in pregnancy. A British Society for Hematology Guideline (2021) [34]	48	48	Not mentioned	Not specified	Not specified	Antenatal, Intrapartum, and Postnatal Care Contraception Counseling and Provision Fertility Care Genetic Counseling	Risk reduction Management
Position statement on the management of pregnancy in sickle cell disease (2025) [The Australian Sickle Cell Disease Working Group] [35]	45	9	Not mentioned	Not specified	Not specified	Antenatal, Intrapartum, and Postnatal Care	Management
Recommendations for Pregnancy in Rare Inherited Anemias (2020) [European Hematology Association] [36]	35	4	GRADE	Strong	High; Moderate	Antenatal, Intrapartum, and Postnatal Care Fertility Care	Screening
Significant haemoglobinopathies: a guideline for screening and diagnosis: a British Society for Hematology guideline (2023) [37]	9	9	GRADE	Strong; Weak	High; Moderate; Low	Antenatal, Intrapartum, and Postnatal Care Contraception Counseling and Provision	Risk reduction Management
Society for Maternal‐Fetal Medicine Consult Series #68: Sickle cell disease in pregnancy (2024) [38]	62	14	Not mentioned	Not specified	Not specified	Fertility Care	Diagnosis Management
Standard of Care Guidelines for Patients with Sickle Cell Disease (2024) [UCSF Center of Excellence Staff] [39]	23	13	GRADE	Weak	Moderate; Low; Very Low	Antenatal, Intrapartum, and Postnatal Care Contraception Counseling and Provision Genetic Counseling	Screening Diagnosis Risk reduction Management
Thalassemias and Sickle Cell Diseases in Pregnancy: SITE Good Practice (2022) [40]	27	27	GRADE	Strong; Weak	Moderate; Low; Very Low	Antenatal, Intrapartum, and Postnatal Care Contraception Counseling and Provision Genetic Counseling	Screening Risk reduction Management
The Diagnosis and Management of Priapism: AUA/SMSNA Guideline (2022) [41]	21	21	GRADE	Not specified	Not specified	Antenatal, Intrapartum, and Postnatal Care Contraception Counseling and Provision	Management

* CPG, clinical practice guideline.

** RHR, reproductive health recommendations.

Approaches to grading recommendations varied across CPGs. Nine guidelines (60%) [30, 31, 33, 36, 39, 40, 42, 43, 44] explicitly reported using the GRADE framework to assess recommendation strength and certainty of evidence, while six (40%) [32, 34, 35, 37, 38, 41] did not report any other formal grading system, such as Oxford, SIGN, or Shekelle Evidence Rating System.

Four CPGs (26.7%) explicitly reported strong recommendations, often alongside weak recommendations within the same guideline [36, 39, 40, 43]. The remaining guidelines (*n* = 8, 53%) did not specify recommendation strength [32, 33, 34, 35, 37, 38, 41, 44]. Regarding certainty of evidence, three CPGs (20%) reported high‐quality evidence [36, 39, 40], and five (33.3%) reported moderate‐quality evidence [36, 39, 40, 42, 43]. Low or very low levels of evidence were frequently reported, often within the same guideline, underscoring limitations in the underlying evidence base. Eight CPGs (53.3%) [32, 33, 34, 35, 37, 38, 41, 44] did not clearly specify the level of evidence supporting their recommendations.

Inclusion of the World Health Organization (WHO) care domains varied. Antenatal, Intrapartum, and Postnatal care were included in 13 CPGs (86.67%) [30, 31, 32, 34, 35, 36, 37, 38, 39, 40, 42, 43, 44], whereas Safe Abortion care was included in only one guideline (6.67%) [36]. At the recommendation level, two‐thirds of all RHRs were classified as Antenatal, Intrapartum, and Postnatal care (*n* = 128, 64.65%), followed by Fertility care (*n* = 37, 18.69%), Genetic Testing (*n* = 16, 8.08%), and Contraception Counseling and Provision (*n* = 16, 8.08%). Safe Abortion Care was explicitly addressed in only one CPG, reflecting a notable gap across reproductive health domains (Figure 2). Detailed findings are illustrated in Additional File 4.

**Figure 2:**
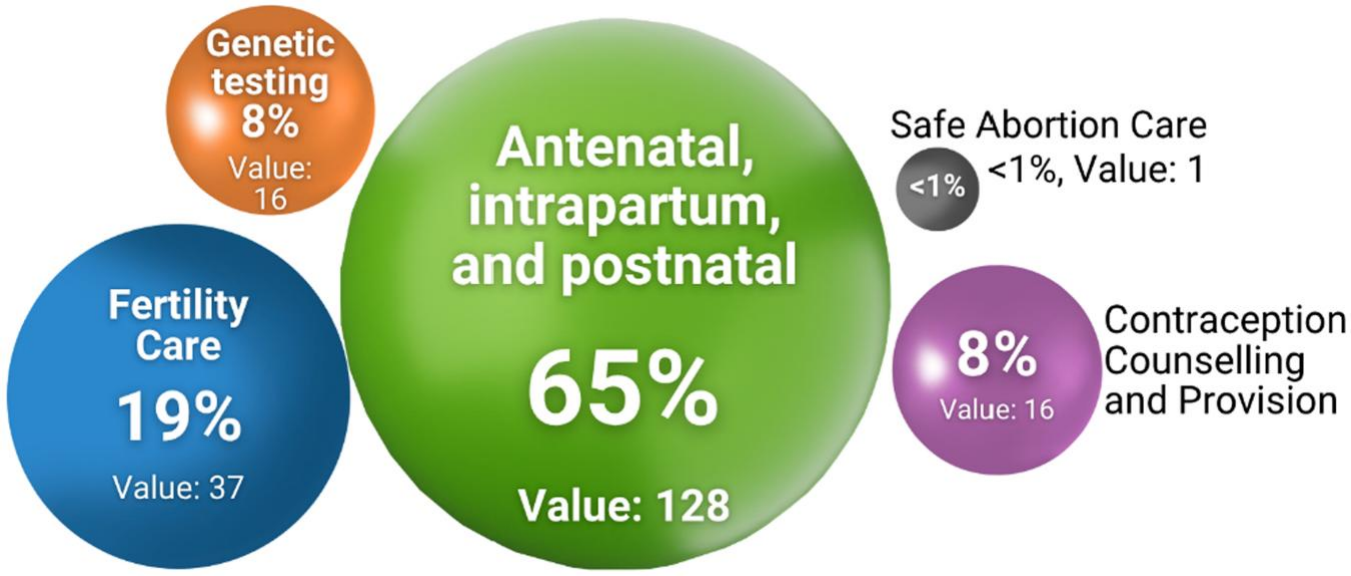
Representation of World Health Organization care domains in reproductive health recommendations (*N* = 198) in included CPGs. *Note:* Percentages rounded to the nearest whole number.

Across the CPGs, stages of care were identified and Management was most often included (*n* = 11, 73.33%) [33, 34, 35, 36, 37, 38, 40, 41, 42, 43, 45], while Diagnosis was infrequently included (*n* = 4, 26.67%) [33, 36, 41, 42], suggesting limited guidance on diagnostic pathways within reproductive health contexts for individuals with SCD. Similarly, at the recommendation level, the stage of care “Management” accounted for the largest proportion of individual RHRs (*n* = 123, 62.12%), followed by Risk Reduction (*n* = 39, 19.70%), Screening (*n* = 24, 12.12%), and Diagnosis (*n* = 12, 6.06%) (Figure 3). See Additional File 5 for detailed findings.

**Figure 3:**
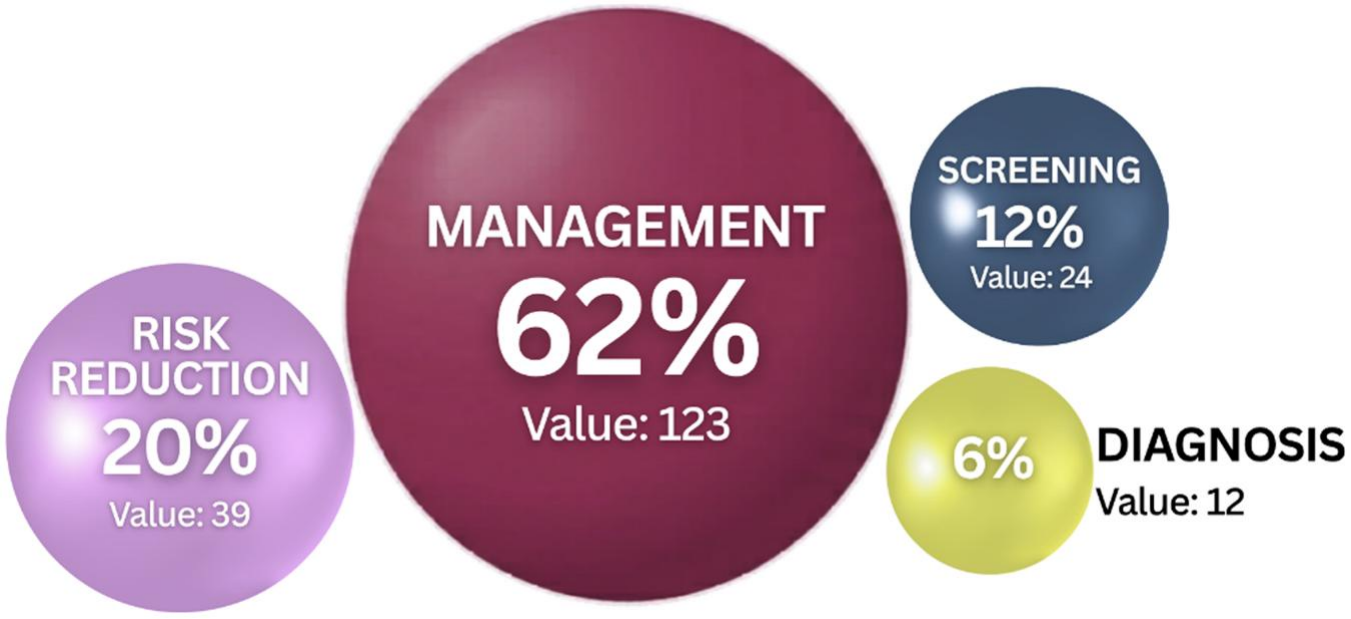
Representation of stages of care in reproductive health recommendations (*N* = 198) in included CPGs. *Note:* Percentages rounded to the nearest whole number.

### 4.4 Quality of Reproductive Health Recommendations (AGREE‐REX)

AGREE‐REX assessment indicated that seven CPGs contained RPHs sufficient overall quality to recommend for use without modification [30, 31, 36, 40, 42, 43, 44], while eight required modifications [32, 33, 34, 35, 37, 38, 39, 41] (see Table 4). Mean scores for Domains 1 (clinical applicability) and 3 (implementability) were both 77%. Across guidelines, the lowest AGREE‐REX score (59%) was observed in Domain 2 (Values and Preferences), indicating limited incorporation of patient and stakeholder perspectives. However, the overall mean AGREE‐REX score across guidelines was 69%. AGREE‐REX scoring demonstrated excellent inter‐rater reliability, with ICCs ranging from 0.85 to 0.95 across domains (see Table 4).

**Table 4:** AGREE‐REX domain scores and recommendations for use.

Guideline	Domain 1 clinical applicability	Domain 2 values and preferences	Domain 3 implementability	Overall	Recommendation for use
American Society of Hematology 2020 guidelines for sickle cell disease: management of acute and chronic pain (2020) [28]	100%	98%	92%	97%	R
American Society of Hematology 2020 guidelines for sickle cell disease: transfusion support (2020) [29]	94%	73%	79%	81%	R
Consensus of the Brazilian Association of Hematology, Hemotherapy and Cellular Therapy (ABHH) and the Brazilian Ministry of Health – General management of blood and blood products on the tests necessary for the release of exceptional medicines for sickle (2024) [30]	56%	52%	58%	55%	RM
First and Second Level Haemoglobinopathies Diagnosis: Best Practices of the Italian Society of Thalassemia and Haemoglobinopathies (SITE) (2022) [31]	72%	38%	54%	53%	RM
Hemoglobinopathies in Pregnancy; Practice Advisory (2025) [American College of Obstetricians and Gynecologists] [32]	50%	35%	75%	49%	RM
Management of acute presentations of sickle cell disease in the emergency department (2024)	64%	58%	63%	61%	RM
[The Royal College of Emergency Medicine] [33]	89%	79%	96%	86%	R
Management of sickle cell disease in pregnancy. A British Society for Hematology Guideline (2021) [34]	69%	48%	79%	62%	RM
Position statement on the management of pregnancy in sickle cell disease (2025) [The Australian Sickle Cell Disease Working Group] [35]	61%	44%	63%	54%	RM
Recommendations for Pregnancy in Rare Inherited Anemias (2020) [European Hematology Association] [36]	61%	58%	58%	59%	RM
Significant haemoglobinopathies: a guideline for screening and diagnosis: a British Society for Hematology guideline (2023) [37]	86%	56%	83%	72%	R
Society for Maternal‐Fetal Medicine Consult Series #68: Sickle cell disease in pregnancy (2024) [38]	69%	38%	71%	56%	RM
Standard of Care Guidelines for Patients with Sickle Cell Disease (2024) [UCSF Center of Excellence Staff] [39]	86%	83%	83%	84%	R
Thalassemias and Sickle Cell Diseases in Pregnancy: SITE Good Practice (2022) [40]	97%	48%	100%	76%	R
The Diagnosis and Management of Priapism: AUA/SMSNA Guideline (2022) [41]	100%	79%	100%	91%	R
Domain mean (SD)	77%	59%	77%	69%	
Standard deviation	17%	19%	16%	16%	
95% Confidence Intervals (CIs) for Mean	67%–86%	48%–69%	68%–85%	60%–77%	
Intraclass Correlation Coefficient (ICC) (between 2 raters)	0.95	0.95	0.85	0.95	

*Notes:* Overall assessment was calculated by averaging all individual items included in the tool.

R, recommended for use; RM, recommended for use with modifications; NR, not recommended for use.

In this dataset, the SDs (ranging from 0.16 to 0.19) are all below 0.25, which suggests moderate spread.

ICCs: calculated absolute ICCs using SPSS v. 32.

## 5 Discussion

This umbrella review evaluated the methodological rigor and content of reproductive health recommendations (RHRs) within clinical practice guidelines (CPGs) for individuals with sickle cell disease (SCD). Overall, the findings show increasing attention to reproductive health in recent guidelines, but also highlight important gaps in consistency, methodological transparency, and coverage of key reproductive health domains.

The attributes of included CPGs reveal that most of the guidelines originated in high‐resource countries, with clear under‐representation of guidelines from regions that carry the highest burden of sickle cell disease (SCD). This imbalance is particularly important given that SCD is predominantly concentrated in low‐ and middle‐income countries (LMICs), especially in sub‐Saharan Africa, where nearly 80% of global cases and most new births occur [46]. Similarly, most children born with SCD and a substantial proportion of disease burden, morbidity, and mortality are concentrated in these settings [2]. Despite this, guideline development remains largely driven by high‐income settings, reflecting differences in research infrastructure, funding availability, and technical capacity for evidence synthesis and guideline production [47]. Recent evidence from LMIC contexts highlights how these structural gaps directly affect care delivery. A 2026 scoping review of SCD care barriers in Africa identified widespread system‐level limitations, including medication stockouts, weak referral systems, and insufficient integration of SCD services across levels of care [48]. In addition, disparities in early diagnosis and access to services remain substantial between HIC and LMIC settings, further reinforcing inequities in care delivery [49]. From the provider's perspective, these gaps are further compounded by limited training and resources. Studies indicate that healthcare systems in LMICs face constraints in workforce capacity, laboratory infrastructure, and access to essential therapies, which restrict the delivery of comprehensive SCD care, including reproductive health services [50]. As a result, critical components such as fertility counseling, contraception, and pregnancy risk management may not be consistently integrated into routine care. This is particularly concerning given that individuals with SCD face significantly increased reproductive risks, including higher maternal morbidity and mortality, adverse pregnancy outcomes, and complex decision‐making related to genetic transmission [51].

Beyond geographic distribution, the results demonstrate considerable heterogeneity in both methodological rigor and the quality of RHRs across CPGs. Although many guidelines reported use of the GRADE framework, inconsistent application and reporting remain in well‐documented challenges. Recent methodological literature highlights that guideline developers do not always apply GRADE criteria consistently, and reporting of recommendation strength and certainty of evidence is often incomplete or unclear [52]. This inconsistency is also reflected in the broader evidence base supporting clinical practice guidelines. For example, a 2025 evaluation of pediatric CPGs found that only 10.6% of recommendations were supported by the highest level of evidence, while a substantial proportion relied on lower‐quality or observational data. In addition, strong and moderate recommendations frequently coexist within the same guideline and may be based on varying levels of evidence, including low‐certainty data, which complicates interpretation and decision‐making in clinical practice [53]. Applying GRADE criteria can be challenging in rare diseases because small and geographically dispersed patient populations can limit recruitment and the feasibility of conducting large randomized controlled trials [54]. Similar challenges related to recruitment, retention, study design, and outcome measurement have been reported in SCD clinical trials [55]. In many LMICs, particularly in sub‐Saharan Africa, historically high childhood mortality among individuals with SCD has likely reduced the population surviving to reproductive age and contributed to the limited evidence available on reproductive health outcomes [56, 57]. Therefore, high‐quality evidence remains limited in areas such as fertility, contraception, and long‐term reproductive outcomes, leading guideline developers to rely on indirect evidence or expert consensus [58]. Consequently, guideline developers may need to rely more heavily on observational studies, indirect evidence, and expert consensus.

Methodological appraisal using AGREE II revealed considerable variation in guideline rigor. While domains related to scope, purpose, and clarity of presentation scored highly, Applicability emerged as a consistent weakness. This pattern is well documented in recent evaluations of clinical practice guidelines, where Scope and Purpose and Clarity of Presentation typically receive the highest scores, while Applicability is often the lowest‐scoring domain [59, 60]. Low scores in this domain suggest that many CPGs fail to provide sufficient guidance on implementation strategies, resource implications, or monitoring and audit criteria that are frequently overlooked during guideline development [59]. Additionally, modest scores in Rigor of Development and Editorial Independence indicate gaps in systematic evidence of synthesis, transparency, and reporting of conflicts of interest. Recent appraisals have similarly identified deficiencies in these domains, particularly related to incomplete reporting of evidence synthesis methods and lack of clarity regarding funding sources and competing interests [61]. These limitations can undermine the credibility and trustworthiness of guidelines, as both methodological rigor and editorial independence are central to ensuring unbiased and evidence‐based recommendations [62].

Assessment of RHRs using AGREE‐REX further demonstrated that fewer than half of the included CPGs contained recommendations of sufficient quality to be adopted without modification. The consistently low scores in the Values and Preferences domain indicate limited explicit consideration of patient perspectives, cultural context, and stakeholder engagement in recommendation development. This finding is supported by recent AGREE‐REX evaluations showing that the Values and Preferences domain is often among the lowest scoring across guidelines, with reported ranges as low as ~45% in some appraisals [63]. Similarly, another recent study has demonstrated that items related to patient values are frequently among the lowest scoring across guidelines [64]. The AGREE‐REX framework explicitly emphasizes that high‐quality recommendations should incorporate patient values and contextual factors to improve their credibility and implementability [64]. This limitation is particularly concerning reproductive health, where shared decision‐making and alignment with patient values are essential. Reproductive health decisions, including contraception, fertility planning, and genetic risk considerations, are preference‐sensitive and require meaningful integration of individual values and sociocultural context [45]. When these factors are not adequately addressed, recommendations may be less acceptable and less likely to be implemented in practice. Furthermore, limited integration of patient‐centered considerations may disproportionately affect historically marginalized and vulnerable populations, including individuals with SCD. Evidence suggests that guideline quality and patient‐centeredness are critical for comprehensive care delivery, particularly in populations facing structural barriers and health disparities [65]. Taken together, these findings underscore the need for guideline development processes that systematically incorporate patient and stakeholder perspectives to enhance the acceptability and real‐world uptake of RHRs.

Analysis of recommendation content showed important gaps across reproductive health domains and stages of care. Although antenatal, intrapartum, and postnatal care were most commonly addressed, other important areas such as contraceptive counseling, fertility care, genetic counseling, and safe abortion care were less frequently included. Recent studies also suggest that reproductive healthcare in SCD is still mainly focused on pregnancy management, with limited attention to fertility counseling, fertility preservation, and comprehensive reproductive planning [15, 66]. In a recent scoping review, including 11 studies, researchers identified two major gaps: limited reproductive healthcare counseling and poor knowledge of disease‐related reproductive risks among adolescents and young adults with SCD. The review found inconsistent counseling regarding fertility risks, contraception, and family‐building options across studies [15]. In addition, fertility preservation and ovarian reserve are becoming more important concerns as survival improves in individuals with SCD, but guidance and evidence in these areas remain limited [67, 68]. In our umbrella review, safe abortion care was included in only one CPG, showing an important gap in comprehensive reproductive health guidance for individuals with SCD, who have high maternal morbidity and mortality risks [51]. Recent literature also emphasizes that reproductive healthcare for individuals with SCD should include comprehensive reproductive counseling and access to the full range of reproductive options. Pecker et al. [69] highlighted that legal restrictions in abortion care may further increase existing challenges in reproductive healthcare for individuals with SCD. The authors also noted that when options such as therapeutic abortion and IVF with preimplantation of genetic testing or are not discussed or accessible, individuals and couples may be denied comprehensive reproductive choices [69]. Therefore, the limited inclusion of safe abortion care in current CPGs may reduce patient‐centered and comprehensive reproductive healthcare for individuals with SCD.

Although this review included guidelines from multiple countries, the findings have important implications for individuals with SCD/T in the United States. Adults with SCD continue to experience challenges related to insurance coverage, transportation, access to specialized care, and continuity of care across the lifespan [70, 71, 72]. These factors may influence timely access to reproductive counseling, preconception care, pregnancy management, and genetic services. Variability in newborn screening follow‐up and communication of SCT results across states may create differences in opportunities for genetic counseling and reproductive health education [73].

The findings are particularly relevant within the current U.S. policy context, where access to contraception, genetic counseling, assisted reproductive technologies, and other services, vary substantially across states. For individuals with SCD, who face increased risks of maternal morbidity and complex reproductive decision‐making—clear, evidence‐based guidance is essential [69]. However, the limited attention given to fertility care, genetic counseling, and safe reproductive care in many existing guidelines may contribute to inconsistency in clinical practice and patient counseling.

Strengthening comprehensive, high‐quality clinical practice guidelines and recommendations across the reproductive lifespan could support more consistent care and informed, patient‐centered decision‐making. As the U.S. healthcare system continues to evolve, improving reproductive health recommendations for individuals with SCD/T may enhance care coordination, expand access to information, and ultimately improve maternal and reproductive health outcomes [70, 72].

Overall, these findings suggest that while reproductive health considerations are increasingly acknowledged with SCD guidelines, existing CPGs vary substantially in rigor, transparency, and patient‐centeredness. Strengthening reproductive health guidance for individuals with SCD will require broader global representation in guideline development, and improved methodological quality, with greater attention to implementation and values‐based decision‐making, as well as concerted efforts to address underrepresented care domains.

## 6 Strengths and Limitations

Using a systematic approach to review CPGs strengthened the rigor and comprehensive synthesis of existing CPGs, minimizing bias and providing a structured overview of the current state of reproductive health guidance for individuals with SCD. The use of predefined methods and guideline appraisal tools (i.e., AGREE II and AGREE‐REX), enabled validated, transparent, reproducible assessments of both methodological rigor and recommendation quality. This review also brings together 198 reproductive health recommendations in a single source, making existing guidance more accessible to healthcare providers, patients, guideline developers, and policymakers. In addition to summarizing available recommendations across the stages and domains of reproductive care, the review identified important gaps and inconsistencies in current guidance. For example, most recommendations focused on antenatal, intrapartum, and postnatal care, whereas safe abortion care was addressed in only one guideline, and guidance on fertility, contraception, genetic counseling, and other reproductive health needs was limited or inconsistently reported. These findings provide new information about areas that are well addressed and those requiring further research, stronger evidence, and future guideline development.

Several limitations should also be acknowledged. First, the review was restricted to CPGs that included RHRs, precluding assessment of the methodological rigor of other SCD CPGs that may indirectly influence reproductive care. Second, the inclusion of consensus‐based CPGs means that some recommendations were primarily informed by expert opinion rather than systematically synthesized evidence, reflecting broader evidence gaps in this field. Third, the review excluded individuals outside the reproductive age range—such as pre‐pubescent children and menopausal and post‐menopausal adults, thus limiting generalizability across the full lifespan of individuals with SCD. Fourth, the review was limited to CPGs published from 2020 onward to capture current clinical guidance. However, this date restriction may have excluded relevant earlier guidelines, including national guidelines developed in LMICs before 2020. Therefore, reproductive health recommendations and perspectives from these settings may be underrepresented in our findings.

Finally, although CPGs published in any language were eligible for inclusion, publication bias is evident, with most identified guidelines originating from high‐ and middle‐income countries and written in English. This likely reflects both reliance on English‐language search strategies and structural barriers faced by low‐resource settings in developing and disseminating formal CPGs. As a result, the findings may not fully capture context‐specific reproductive health needs or best practices in regions most affected by SCD.

## 7 Conclusion

This systematic review demonstrates that while reproductive health is increasingly acknowledged with clinical practice guidelines for sickle cell disease, significant gaps remain in both the quality and applicability of existing guidance. Most identified CPGs originated from high‐resource settings, highlighting a geographic mismatch between guideline development and the global burden of SCD. Although many guidelines addressed antenatal, intrapartum, and postnatal care, other essential reproductive health domains—particularly contraception, fertility care, genetic counseling, and safe abortion care—were inconsistently included or absent altogether.

Methodological appraisal revealed wide variability in rigor, transparency, and implementation of readiness. Limitations in applicability, editorial independence, and the incorporation of patient values and preferences suggest that many guidelines may be difficult to operationalize and may be insufficiently responsive to the needs of individuals with SCD. Furthermore, the predominance of low or very low certainty of evidence underscores knowledge gaps and the reliance on expert consensus in shaping RHRs.

Collectively, these findings point to an urgent need for the development of high‐quality, globally inclusive, and patient‐centered CPGs that address the full reproductive life course of individuals with SCD. Future guideline efforts should prioritize methodological rigor, stakeholder engagement, and contextual adaptability, while at the same time, investments in primary research are needed to strengthen the evidence base underpinning reproductive health care in the population. Advancing these priorities is essential to improving reproductive health outcomes and supporting informed, shared decision‐making for individuals living with sickle cell disease.

## Author Contributions

**Lisa R. Roberts:** conceptualization, methodology, software, data curation, investigation, validation, supervision, funding acquisition, visualization, project administration, resources, writing – original draft, writing – review and editing. **Marc Julmisse:** data curation, validation, formal analysis, visualization, writing – review and editing, writing – original draft. **Safiye Sahin:** methodology, software, data curation, investigation, validation, formal analysis, visualization, writing – original draft, writing – review and editing. **Jan M. Nick:** conceptualization, methodology, software, data curation, investigation, validation, formal analysis, supervision, visualization, writing – original draft, writing – review and editing. **Shanalee Tamares:** writing – review and editing, software, investigation, validation, visualization, writing – original draft.

## Policy on Using ChatGPT and Similar AI Tools

The authors declare use of AI (ChatGPT v. 4.5) for point clarifications, tutoring, alternate phrase suggestions, and grammatical refinements. Original thinking, intellectual contributions, and the entire construction of the protocol was done solely by the authors.

## Ethics Statement

The authors have nothing to report.

## Consent

The authors have nothing to report.

## Conflicts of Interest

The authors declare no conflicts of interest.

## Supporting information


Supporting File 1

Supporting File 2

Supporting File 3

Supporting File 4

Supporting File 5

## Data Availability

The data that support the findings of this study are available from the corresponding author upon reasonable request.
