# Supplementary material for: Evaluating Reproductive Health Recommendations in CPGs for Sickle Cell Disease: An Umbrella Review
Source: Clin Public Health Guidel. 2026 Sep 22;3(4):e70091. doi: 10.1002/gin2.70091 (PMC13618671; doi:10.1002/gin2.70091)
Supplement: Supplementary file 1 [file GIN2-3-e70091-s001.docx]

**Appendix I. Search Strategy**

**Databases**

| Embase (Embase.com)  Date searched: March 13, 2025 | ('sickle cell anemia'/mj OR 'hb ss disease':ti,ab OR 'ss disease (sickle cell)':ti,ab OR 'anaemia, sickle cell':ti,ab OR 'anemia, sickle cell':ti,ab OR 'haemoglobin ss':ti,ab OR 'haemoglobin ss disease':ti,ab OR 'hemoglobin ss':ti,ab OR 'hemoglobin ss disease':ti,ab OR 'homozygous hb s disease':ti,ab) AND ('clinical practice guideline'/mj OR 'clinical practice guideline':ti,ab OR 'clinical practice guidelines':ti,ab OR 'practice guideline'/mj OR 'practice guideline':ti,ab OR 'practice guidelines':ti,ab OR 'clinical guideline':ti,ab OR 'clinical guidelines':ti,ab OR 'clinical pathway'/mj OR 'care pathway':ti,ab OR 'management protocol':ti,ab OR 'clinical pathways':ti,ab OR 'care pathways':ti,ab OR 'management protocols':ti,ab OR 'critical pathway':ti,ab OR 'critical pathways':ti,ab OR 'treatment protocol'/mj OR 'treatment protocols':ti,ab OR 'clinical recommendations':ti,ab OR 'clinical recommendation':ti,ab OR 'practice recommendation':ti,ab OR 'practice recommendations':ti,ab OR 'guideline recommendation':ti,ab OR 'guideline recommendations':ti,ab) | 294 |
| --- | --- | --- |
| Pubmed (NCBI)  Date searched: March 12, 2025 | ("anemia, sickle cell"[MeSH Terms] OR "sickle cell anemia"[tiab] OR "sickle cell disease"[tiab] OR "Hb SS disease"[tiab] OR "SS disease"[tiab] OR "haemoglobin SS"[tiab] OR "hemoglobin SS"[tiab] OR "homozygous Hb S disease"[tiab]) AND ("Guideline"[pt] OR "practice guideline"[pt] OR "consensus development conference"[pt] OR "consensus development conference, NIH"[pt] OR guideline*[ti] OR standards[ti] OR consensus*[ti] OR recommendat*[ti] OR guideline*[cn] OR standards[cn] OR consensus*[cn] OR recommendat*[cn] OR "practice parameter*"[ti] OR "position statement*"[ti] OR "practice bulletin*"[ti] OR "policy statement*"[ti] OR CPG[ti] OR CPGs[ti] OR "best practice*"[ti] OR (care[ti] AND (path[ti] OR paths[ti] OR pathway[ti] OR pathways[ti] OR map[ti] OR maps[ti] OR plan[ti] OR plans[ti] OR standard[ti])) OR ((critical[ti] OR clinical[ti] OR practice[ti]) AND (path[ti] OR paths[ti] OR pathway[ti] OR pathways[ti] OR protocol*[ti])) OR (algorithm*[ti] AND (pharmacotherap*[ti] OR chemotherap*[ti] OR chemotreatment*[ti] OR therap*[ti] OR treatment*[ti] OR intervention*[ti])) OR (algorithm*[ti] AND (screening[ti] OR examination[ti] OR test[ti] OR tested[ti] OR testing[ti] OR assessment*[ti] OR diagnosis[ti] OR diagnoses[ti] OR diagnosed[ti] OR diagnosing[ti])) OR guideline*[ot] OR standards[ot] OR consensus*[ot] OR recommendat*[ot] OR "practice parameter*"[ot] OR "position statement*"[ot] OR "practice bulletin*"[ot] OR "policy statement*"[ot] OR CPG[ot] OR CPGs[ot] OR "best practice*"[ot] OR (care[ot] AND (path[ot] OR paths[ot] OR pathway[ot] OR pathways[ot] OR map[ot] OR maps[ot] OR plan[ot] OR plans[ot] OR standard[ot])) OR ((critical[ot] OR clinical[ot] OR practice[ot]) AND (path[ot] OR paths[ot] OR pathway[ot] OR pathways[ot] OR protocol*[ot])) OR (algorithm*[ot] AND (pharmacotherap*[ot] OR chemotherap*[ot] OR chemotreatment*[ot] OR therap*[ot] OR treatment*[ot] OR intervention*[ot])) OR (algorithm*[ot] AND (screening[ot] OR examination[ot] OR test[ot] OR tested[ot] OR testing[ot] OR assessment*[ot] OR diagnosis[ot] OR diagnoses[ot] OR diagnosed[ot] OR diagnosing[ot])) OR (("Systematic review"[ti] OR "systematic review"[pt] OR "systematic review"[ot]) AND ("practice guideline*"[tiab] OR "treatment guideline*"[tiab] OR "clinical guideline*"[tiab] OR "guideline recommendation*"[tiab]))) | 371 |
| CINAHL (EBSCO)  Date searched: March 14, 2025 | AB (MH "Anemia, Sickle Cell+" OR "sickle cell anemia" OR "sickle cell disease" OR "Hb SS disease" OR "SS disease" OR "haemoglobin SS" OR "hemoglobin SS" OR "homozygous Hb S disease") AND AB (MH "Practice Guidelines" OR "practice guideline*" OR MH "Critical Path" OR "clinical practice guideline*" OR "clinical guideline*" OR MH "Protocols+" OR "consensus statement*" OR "consensus development" OR "best practice*" OR "care standard*" OR "standard* of care" OR "clinical pathway*" OR "care pathway*" OR "integrated care pathway*" OR "critical pathway*" OR "care map*" OR "clinical protocol*" OR "treatment protocol*" OR "care protocol*" OR "management protocol*" OR "clinical recommendation*" OR "practice recommendation*" OR "evidence-based recommendation*" OR "guideline recommendation*") | 293 |
| PsycInfo (EBSCO)  Date searched: March 13, 2025 | AB (DE "Sickle Cell Disease" OR "sickle cell anemia" OR "sickle cell disease" OR "Hb SS disease" OR "SS disease" OR "haemoglobin SS" OR "hemoglobin SS" OR "homozygous Hb S disease") AND AB (DE "Best Practices" OR guideline* OR "practice guideline*" OR "clinical practice guideline*" OR "clinical guideline*" OR "consensus statement*" OR "consensus development" OR "best practice*" OR "care standard*" OR "standard* of care" OR pathway* OR "clinical pathway*" OR "care pathway*" OR "integrated care pathway*" OR "critical pathway*" OR "care map*" OR protocol* OR "clinical protocol*" OR "treatment protocol*" OR "care protocol*" OR "management protocol*" OR "clinical recommendation*" OR "practice recommendation*" OR "evidence-based recommendation*" OR "guideline recommendation*") | 116 |
| Cochrane Library (Cochrane Reviews) (Wiley)  Date searched: March 13, 2025 | ("sickle cell anemia" OR "sickle cell disease" OR "Hb SS disease" OR "SS disease" OR "haemoglobin SS" OR "hemoglobin SS" OR "homozygous Hb S disease") AND (guideline* OR "practice guideline" OR “practice guidelines” OR "clinical practice guideline" OR “clinical practice guidelines” OR "clinical guideline" OR “clinical guidelines” OR "consensus statement" OR "consensus development" OR "best practice" OR “best practices” OR "care standard" OR "standard of care" OR “standards of care” OR pathway* OR "clinical pathway" OR “clinical pathways” OR "care pathway" OR “care pathways” OR "integrated care pathway" OR “integrated care pathways” OR "critical pathway" OR “critical pathways” OR "care map" OR protocol* OR "clinical protocol" OR “clinical protocols” OR "treatment protocol" OR “treatment protocols” OR "care protocol" OR “care protocols” OR "management protocol" OR “management protocols” OR "clinical recommendation" OR “clinical recommendations” OR "practice recommendation" OR “practice recommendations” OR "evidence-based recommendation" OR "guideline recommendations" OR "evidence-based recommendations" OR "guideline recommendations") | 12 |
| DynaMed (EBSCO)  Date searched: April 30, 2025 | Sickle Cell Disease in Adults and Adolescents (Condition) > Guidelines | 27 |
| TripPro (Trip Database Ltd.)  Date searched: March 13, 2025 | (title:"sickle cell anemia" OR title:"sickle cell disease" OR title:"Hb SS disease" OR title:"SS disease" OR title:"haemoglobin SS" OR title:"hemoglobin SS" OR title:"homozygous Hb S disease") AND ("practice guideline" OR "practice guidelines" OR "clinical practice guideline" OR "clinical practice guidelines" OR "clinical guideline" OR "clinical guidelines" OR "consensus statement" OR "consensus development" OR "best practice" OR "best practices" OR "care standard" OR "standard of care" OR "standards of care" OR "clinical pathway" OR "clinical pathways" OR "care pathway" OR "care pathways" OR "integrated care pathway" OR "integrated care pathways" OR "critical pathway" OR "critical pathways" OR "care map" OR "clinical protocol" OR "clinical protocols" OR "treatment protocol" OR "treatment protocols" OR "care protocol" OR "care protocols" OR "management protocol" OR "management protocols" OR "clinical recommendation" OR "clinical recommendations" OR "practice recommendation" OR "practice recommendations" OR "evidence-based recommendation" OR "guideline recommendations" OR "evidence-based recommendations" OR "guideline recommendations") | 239 |
| Web of Science Core Collection (Clarivate)  Date searched: March 13, 2025 | ("sickle cell anemia" OR "sickle cell disease" OR "Hb SS disease" OR "SS disease" OR "haemoglobin SS" OR "hemoglobin SS" OR "homozygous Hb S disease") (Title) and ("practice guideline" OR "practice guidelines" OR "clinical practice guideline" OR "clinical practice guidelines" OR "clinical guideline" OR "clinical guidelines" OR "consensus statement" OR "consensus development" OR "best practice" OR "best practices" OR "care standard" OR "standard of care" OR "standards of care" OR "clinical pathway" OR "clinical pathways" OR "care pathway" OR "care pathways" OR "integrated care pathway" OR "integrated care pathways" OR "critical pathway" OR "critical pathways" OR "care map" OR "clinical protocol" OR "clinical protocols" OR "treatment protocol" OR "treatment protocols" OR "care protocol" OR "care protocols" OR "management protocol" OR "management protocols" OR "clinical recommendation" OR "clinical recommendations" OR "practice recommendation" OR "practice recommendations" OR "evidence-based recommendation" OR "guideline recommendations" OR "evidence-based recommendations" OR "guideline recommendations") (Abstract) | 216 |
| Google Scholar  Date searched: March 26, 2025 | ("sickle cell anemia"\|"sickle cell disease"\|"Hb SS disease")("clinical practice guideline"\|"practice guideline"\|"clinical guideline"\|"consensus statement"\|"best practice"\|"standard of care"\|"clinical pathway"\|"clinical protocol"\|"clinical recommendation") | 1^st^ 200 |
| American society of hematology <https://www.hematology.org>  Date searched: April 15, 2025 | site:https://www.hematology.org ((guideline\|protocol\|pathway)sickle\|HbSS) | 8 |
| CDC  <https://www.cdc.gov/>  Date searched: April 14, 2025 | site:https://www.cdc.gov/ ((guideline\|protocol\|pathway)sickle\|HbSS) | 21 |
| National Heart, Lung, and Blood Institute  s<https://www.nhlbi.nih.gov/>  Date searched: April 15, 2025 | site:<https://www.nhlbi.nih.gov/> ((guideline\|protocol\|pathway)sickle\|HbSS) | 2 |
| National Institutes of Health  <https://www.nhlbi.nih.gov/>  Date searched: April 14, 2025 | site:<https://www.nhlbi.nih.gov/> ((guideline\|protocol\|pathway)sickle\|HbSS) | 3 |
| Guideline Central  <https://www.guidelinecentral.com/guidelines/>  Date searched: April 14, 2025 | site:https://www.guidelinecentral.com/guidelines/ ((guideline\|protocol\|pathway)sickle\|HbSS) | 21 |
| ECRI Guidelines Trust  <https://guidelines.ecri.org/>  Date searched: April 14, 2025 | site:<https://guidelines.ecri.org/> ((guideline\|protocol\|pathway)sickle\|HbSS) | 15 |
| American Academy of Pediatrics  <https://www.aap.org/en/>  Date searched: April 23, 2025 | site:https://www.aap.org/en/ ((guideline\|protocol\|pathway)sickle\|HbSS) | 0 |
| American College of Physicians Clinical Practice Guidelines  <https://www.acponline.org/clinical-information/guidelines>  Date searched: April 23, 2025 | site:<https://www.acponline.org/clinical-information/guidelines> ((guideline\|protocol\|pathway)sickle\|HbSS) | 0 |
| US Department of Health and Human Services  <https://health.gov/healthypeople/tools-action/browse-evidence-based-resources>  Date searched: April 23, 2025 | site:<https://health.gov/healthypeople/tools-action/browse-evidence-based-resources> ((guideline\|protocol\|pathway)sickle\|HbSS) | 1 |
| National Guideline Clearinghouse  http://www.guidelines.gov/  Date searched: April 24, 2025 | site:http://www.guidelines.gov/ ((guideline\|protocol\|pathway)sickle\|HbSS) | 0 |
| BC Clinical Practice Guidelines & Protocols  <https://www2.gov.bc.ca/gov/content/health/practitioner-professional-resources/bc-guidelines>  Date searched: April 24, 2025 | site:https://www2.gov.bc.ca/gov/content/health/practitioner-professional-resources/bc-guidelines ((guideline\|protocol\|pathway)sickle\|HbSS) | 0 |
| College of Family Physicians of Canada  <https://www.cfpc.ca/en/home>  Date searched: April 24, 2025 | site:https://www.cfpc.ca/en/home ((guideline\|protocol\|pathway)sickle\|HbSS) | 0 |
| Health Canada - Practice Guidelines  <https://www.canada.ca/en/health-canada.html>  Date searched: April 24, 2025 | site:https://www.canada.ca/en/health-canada.html ((guideline\|protocol\|pathway)sickle\|HbSS | 0 |
| Registered Nurses Association of Ontario's Best Practice Guidelines  <https://rnao.ca/bpg/guidelines>  Date searched: April 24, 2025 | site:https://rnao.ca/bpg/guidelines ((guideline\|protocol\|pathway)sickle\|HbSS) | 0 |
| National Institute for Health Care Excellence (NICE)  <https://www.nice.org.uk/Guidance>  Date searched: April 25, 2025 | site:https://www.nice.org.uk/Guidance (("sickle"\|HbSS)(guideline\|protocol\|pathway)) | 10 |
| Scottish Intercollegiate Guidelines Network (SIGN)  <https://www.sign.ac.uk/our-guidelines/>  Date searched: April 30, 2025 | site:<https://www.sign.ac.uk/our-guidelines/> (("sickle"\|HbSS)(guideline\|protocol\|pathway)) | 0 |
