# Supplementary material for: Evaluating Reproductive Health Recommendations in CPGs for Sickle Cell Disease: An Umbrella Review
Source: Clin Public Health Guidel. 2026 Sep 22;3(4):e70091. doi: 10.1002/gin2.70091 (PMC13618671; doi:10.1002/gin2.70091)
Supplement: Supplementary file 2 [file GIN2-3-e70091-s003.docx]

**Appendix II. Excluded studies**

1. Abdel-Hadi L, Carmenate YV, Castillo-Aleman YM, Sheikh S, Zakaria A, Phillips J. Treatment of sickle cell disease-options and perspective. Am J Blood Res. 2023;13(2):61. Available from: https://pmc.ncbi.nlm.nih.gov/articles/PMC10195315/pdf/ajbr0013-0061.pdf

**Reason for exclusion: Ineligible study design**

2. Alanazi N. Clinical guidelines for management of acute painful episodes of vaso-occlusive crisis in children with sickle cell disease. Adv Life Sci. 2021;8(3):221–7.

**Reason for exclusion: No Reproductive health recommendations**

3. Alashkar F, Aramayo-Singelmann C, Böll J, Hoferer A, Jarisch A, Kamal H, et al. Transition in sickle cell disease (SCD): A German consensus recommendation. J Pers Med. 2022;12(7):9. doi:10.3390/jpm12071156

**Reason for exclusion: Ineligible study design**

4. American Society of Hematology. Plan for New ASH Clinical Practice Guidelines on dosing and monitoring of hydroxyurea for sickle cell disease. [date unknown].

**Reason for exclusion: Ineligible study design**

5. Anderson DR, Morgano GP, Bennett C, Dentali F, Francis CW, Garcia DA, et al. American Society of Hematology 2019 guidelines for management of venous thromboembolism: prevention of venous thromboembolism in surgical hospitalized patients. Blood Adv. 2019;3(23):3898–944. doi:10.1182/ bloodadvances.2019000975.

**Reason for exclusion: No Reproductive health recommendations**

6. American Society of Hematology. ASH clinical practice guidelines on sickle cell disease (SCD). American Society of Hematology. 2021. Available from: https://www.hematology.org/-/media/hematology/images/clinicians/guidelines/ashnew2021ashclinicalpracticeguidelinesonscd92721.pdf?la=en&hash=B1E96167DBDC5182FA032D5D6D911B5B

**Reason for exclusion: No Reproductive health recommendations**

7. The Hospital for Sick Children. Acute chest syndrome or pneumonia: guidelines for management in children with sickle cell disease. SickKids Supportive Care Guidelines. 2021. Available from: https://wapps.sickkids.ca/clinical-practice-guidelines/clinical-practice-guidelines/Export/CLINS216/Main%20Document.pdf

**Reason for exclusion: No Reproductive health recommendations**

8. The Hospital for Sick Children. Acute painful episodes vaso-occlusive crisis: guidelines for management in children with Sickle Cell Disease. SickKids Supportive Care Guidelines. 2021. Available from: https://wapps.sickkids.ca/clinical-practice-guidelines/clinical-practice-guidelines/Export/CLINS214/Main%20Document.pdf

**Reason for exclusion: No Reproductive health recommendations**

9. The Hospital for Sick Children. Acute Splenic Sequestration: Guidelines for management in children with sickle cell disease. SickKids Supportive Care Guidelines. 2021. Available from: https://wapps.sickkids.ca/clinical-practice-guidelines/clinical-practice-guidelines/Export/CLINS218/Main%20Document.pdf

**Reason for exclusion: No Reproductive health recommendations**

10. Kanter J, Liem RI, Bernaudin F, Bolanos-Meade J, Fitzhugh CD, Hankins JS, et al. American Society of Hematology 2021 guidelines for sickle cell disease: stem cell transplantation. Blood Adv. 2021;5(18):3668–89. doi:10.1182/bloodadvances.2021004394C

**Reason for exclusion: Duplicate study**

11. The Hospital for Sick Children. Fever: guidelines for management in children with sickle cell disease. SickKids Supportive Care Guidelines. 2021. Available from: https://wapps.sickkids.ca/clinical-practice-guidelines/clinical-practice-guidelines/Export/CLINS215/Main%20Document.pdf

**Reason for exclusion: No Reproductive health recommendations**

12. Driggers RW, Bryant AS, Ghidini A. Indications for outpatient antenatal fetal surveillance: ACOG Committee Opinion, Number 828. Obstet Gynecol. 2021;137(6):e177–e97. doi:10.1097/AOG.0000000000004407

**Reason for exclusion: Ineligible study design**

13. Opioid prescribing guideline. Prim Care Drug Alerts. 2022;43(12):46–7.

**Reason for exclusion: Not available through ILL**

14. The Hospital for Sick Children. Peri-operative management: guidelines for in-patient management of children with sickle cell disease. SickKids Supportive Care Guidelines. 2021. Available from: https://wapps.sickkids.ca/clinical-practice-guidelines/clinical-practice-guidelines/Export/CLINS221/Main%20Document.pdf

**Reason for exclusion: No Reproductive health recommendations**

15. Jean LS. Sickle cell disease obstetric multi-disciplinary care programme. In: ClinicalTrials.gov. 2022; Available from: https://clinicaltrials.gov/study/NCT05143021

**Reason for exclusion: Ineligible study design**

16. The Hospital for Sick Children. Sickle cell disease: guidelines to hydroxyurea. SickKids Supportive Care Guidelines. 2024. Available from: https://wapps.sickkids.ca/clinical-practice-guidelines/clinical-practice-guidelines/Export/CLINS224/Main%20Document.pdf

**Reason for exclusion: No Reproductive health recommendations**

17. The Hospital for Sick Children. Stroke: guidelines for in-patient management in children with sickle cell disease. SickKids Supportive Care Guidelines. 2021. Available from: https://wapps.sickkids.ca/clinical-practice-guidelines/clinical-practice-guidelines/Export/CLINS219/Main%20Document.pdf

**Reason for exclusion: No Reproductive health recommendations**

18. Commissioners SC. The Lancet Haematology Commission on sickle cell disease: key recommendations. Lancet Haematol. 2023;10(8):e564–7. doi:10.1016/S2352-3026(23)00154-0

**Reason for exclusion: Ineligible study design**

19. Brandow AM, Carroll CP, Creary S, Edwards-Elliott R, Glassberg J, Hurley RW, et al. American Society of Hematology 2020 guidelines for sickle cell disease: management of acute and chronic pain. Blood Adv. 2020;4(12):2656–701. doi:10.1182/bloodadvances.2020001851.

**Reason for exclusion: Duplicate study**

20. Centers for Disease Control and Prevention. Prevention and treatment of SCD complications. 2024. Available from: https://www.cdc.gov/sickle-cell/about/prevention-and-treatment.html

**Reason for exclusion: Ineligible study design**

21. Centers for Disease Control and Prevention. Women with sickle cell disease and postpartum care: what to know after delivering your baby. 2024. Available from: https://www.cdc.gov/sickle-cell/media/fact-sheets/pregnancy/women-with-scd-and-postpartum-care.pdf

**Reason for exclusion: Ineligible study design**

22. Chakravorty S, Anie KA, Dziwinski S, Kaya B, Green E, Millanaise S, et al. Optimising the transition from paediatric to adult care model for people with sickle cell disease – a UK multidisciplinary consensus statement. Health Sci J. 2019;13(3):1–10.

**Reason for exclusion: No Reproductive health recommendations**

23. Chou ST, Alsawas M, Fasano RM, Field JJ, Hendrickson JE, Howard J, et al. American Society of Hematology 2020 guidelines for sickle cell disease: transfusion support. Blood Adv. 2020;4(2):327–55. doi:10.1182/bloodadvances.2019001143.

**Reason for exclusion: Duplicate study**

24. DeBaun MR, Jordan LC, King AA, Schatz J, Vichinsky E, Fox CK, et al. American Society of Hematology 2020 guidelines for sickle cell disease: prevention, diagnosis, and treatment of cerebrovascular disease in children and adults. Blood Adv. 2020;4(8):1554–88. doi: 10.1182/bloodadvances.2019001142

**Reason for exclusion: No Reproductive health recommendations**

25. Management of acute events of sickle cell disease. In: DynaMed [database on the Internet]. 2022. Available from <https://www.dynamed.com/management/management-of-acute-events-of-sickle-cell-disease> Subscription required to view.

**Reason for exclusion: Ineligible study design**

26. Management of Sickle Cell Disease in Pregnancy. In: DynaMed [database on the Internet]. 2023. Available from <https://www.dynamed.com/management/management-of-sickle-cell-disease-in-pregnancy> Subscription required to view.

**Reason for exclusion: Ineligible study design**

27. Holvoet L, Keohavong A, Traore K. Care pathway for the sickle cell child. Rev Infirm. 2022;71(277):18–9. doi:10.1016/j.revinf.2021.11.004

**Reason for exclusion: Not available through ILL**

28. Izcovich A, Cuker A, Kunkle R, Neumann I, Panepinto J, Pai M, et al. A user guide to the American Society of Hematology clinical practice guidelines. Blood Adv. 2020;4(9):2095–110. doi:10.1182/bloodadvances.2020001755.

**Reason for exclusion: Ineligible study design**

29. Kanter J, Liem RI, Bernaudin F, Bolaños-Meade J, Fitzhugh CD, Hankins JS, et al. American Society of Hematology 2021 guidelines for sickle cell disease: stem cell transplantation. Blood Adv. 2021;5(18):3668–89. doi:10.1182/bloodadvances.2021004394C

**Reason for exclusion: No Reproductive health recommendations**

30. Kleindorfer DO, Towfighi A, Chaturvedi S, Cockroft KM, Gutierrez J, Lombardi-Hill D, et al. 2021 guideline for the prevention of stroke in patients with stroke and transient ischemic attack: a guideline from the American Heart Association/American Stroke Association. Stroke. 2021 Jul;52(7):e364-467 doi:10.1161/STR.0000000000000375

**Reason for exclusion: No Reproductive health recommendations**

31. Liem RI, Lanzkron S, Coates TD, DeCastro L, Desai AA, Ataga KI, et al. American Society of Hematology 2019 guidelines for sickle cell disease: cardiopulmonary and kidney disease. Blood Adv. 2019 Dec 10;3(23):3867-97. doi:10.1182/ bloodadvances.2019000916.

**Reason for exclusion: Duplicate study**

32. Borges EL, Spira JA, Garcia TF. Recommendations for the management of leg ulcers in people with sickle cell disease. Rev Enferm UERJ. 2020;28:e50170. doi:10.12957/reuerj.2020.50170

**Reason for exclusion: Ineligible study design**

33. Lobitz S, Cario H. Recommendations for action according to the sickle cell disease guideline Pain crises in patients with sickle cell disease. Monatsschr Kinderheilkd. 2022 Nov 1;170(11):1034-7. doi:10.1007/s00112-022-01467-y

**Reason for exclusion: No Reproductive health recommendations**

34. Loggetto SR, de Almeida Veríssimo MP, Darrigo-Junior LG, dos Santos Simoes R, Bernardo WM, Braga JA. Guidelines on sickle cell disease: primary stroke prevention in children and adolescents. Hematol Transfus Cell Ther. 2022;44(1):85–94. doi:10.1016/j.htct.2021.09.013

**Reason for exclusion: No Reproductive health recommendations**

35. Lyman GH, Carrier M, Ay C, Di Nisio M, Hicks LK, Khorana AA, et al. American Society of Hematology 2021 guidelines for management of venous thromboembolism: prevention and treatment in patients with cancer. Blood Adv. 2021 Feb 23;5(4):927-74. doi:10.1182/bloodadvances.2020003442.

**Reason for exclusion: Ineligible participant characteristics**

36. Maitland K, Kiguli S, Olupot‐Olupot P, Opoka RO, Chimalizeni Y, Alaroker F, et al. Transfusion management of severe anaemia in African children: a consensus algorithm. Br J Haematol. 2021;193(6):1247–59. doi:10.1111/bjh.17429

**Reason for exclusion: No Reproductive health recommendations**

37. Mbaeyi S. The Advisory Committee on Immunization Practices’ interim recommendations for additional primary and booster doses of COVID-19 vaccines — United States, 2021. MMWR morb Mortal Wkly Rep. 2021;70(44):1545-1552. Available from: https://www.cdc.gov/mmwr/volumes/70/wr/mm7044e2.htm?ftag=MSF0951a18

**Reason for exclusion: Ineligible participant characteristics**

38. Middeldorp S, Nieuwlaat R, Baumann Kreuziger L, Coppens M, Houghton D, James AH, et al. American Society of Hematology 2023 guidelines for management of venous thromboembolism: thrombophilia testing. Blood Adv. 2023;7(22):7101–38. doi:10.1182/bloodadvances.2023010177

**Reason for exclusion: Ineligible participant characteristics**

39. Montironi R, Cupaiolo R, Kadji C, Badr DA, Deleers M, Charles V, et al. Management of sickle cell disease during pregnancy: experience in a third-level hospital and future recommendations. J Matern Fetal Neonatal Med. 2022;35(12):2345–54. doi:10.1080/14767058.2020.1786054

**Reason for exclusion: Ineligible study design**

40. Murad MH, Liem RI, Lang ES, Akl EA, Meerpohl JJ, DeBaun MR, et al. 2019 sickle cell disease guidelines by the American Society of Hematology: methodology, challenges, and innovations. Blood Adv. 2019;3(23):3945–50. doi:10.1182/bloodadvances.2019000931.

**Reason for exclusion: Ineligible study design**

41. Neumann I, Izcovich A, Aguilar R, Basantes GL, Casais P, Colorio CC, et al. American Society of Hematology, ABHH, ACHO, Grupo CAHT, Grupo CLAHT, SAH, SBHH, SHU, SOCHIHEM, SOMETH, Sociedad Panameña de Hematología, Sociedad Peruana de Hematología, and SVH 2022 guidelines for prevention of venous thromboembolism in surgical and medical patients and long-distance travelers in Latin America. Blood Adv. 2022;6(12):3636–49. doi:10.1182/bloodadvances.2021006482

**Reason for exclusion: Ineligible participant characteristics**

42. Nguyen AT. US medical eligibility criteria for contraceptive use, 2024. MMWR. Recomm Rep. 2024;73(4):1–126. Available from: https://www.cdc.gov/mmwr/volumes/73/rr/rr7304a1_appendix.htm

**Reason for exclusion: Ineligible study design**

43. Han H, Hensch L, Tubman VN. Indications for transfusion in the management of sickle cell disease. Hematol. 2021;2021(1):696–703. doi:10.1182/hematology.2021000307

**Reason for exclusion: Ineligible study design**

44. Alashkar F, Aramayo-Singelmann C, Böll J, Hoferer A, Jarisch A, Kamal H, et al. Transition in Sickle Cell Disease (SCD): A German consensus recommendation. J Pers Med. 2022;12(7):9. doi: 10.3390/jpm12071156

**Reason for exclusion: Duplicate study**

45. Böll J, Alashkar F, Aramayo-Singelmann C, Hoferer A, Jarisch A, Kamal H, et al. [Transition in sickle cell disease - recommendations of the transition initiative sickle cell disease]. Dtsch Med Wochenschr. 2024;149(10):579–86. doi:10.1055/a-2252-8113

**Reason for exclusion: Ineligible study design**

46. Ogu UO, Mukhopadhyay A, Patel K, Nelson MN, Strahan KS, Wu L, et al. Hydroxyurea at escalated dose versus fixed low-dose hydroxyurea in adults with sickle cell disease. Eur J Haematol. 2024;112(3):466–74. doi:10.1111/ejh.14138

**Reason for exclusion: Ineligible study design**

47. Ortel TL, Neumann I, Ageno W, Beyth R, Clark NP, Cuker A, et al. American Society of Hematology 2020 guidelines for management of venous thromboembolism: treatment of deep vein thrombosis and pulmonary embolism. Blood Adv. 2020;4(19):4693–738. doi:10.1182/bloodadvances.2020001830.

**Reason for exclusion: Ineligible participant characteristics**

48. Paintsil V, Ally M, Isa H, Anie KA, Mgaya J, Nkanyemka M, et al. Development of multi-level standards of care recommendations for sickle cell disease: Experience from SickleInAfrica. Front Genet. 2023;13:1052179. doi:10.3389/fgene.2022.1052179

**Reason for exclusion: Ineligible study design**

49. Pecker LH, Cameron K. Sickle cell disease and infertility risks: implications for counseling and care of affected girls and women. Expert Rev Hematol. 2024;17(8):493–504. doi:0.1080/17474086.2024.2372320.

**Reason for exclusion: Ineligible study design**

50. Pecker LH, Lanzkron S. Sickle cell disease. Ann Intern Med. 2021;174(1):ITC1-ITC16. doi:10.7326/AITC202101190

**Reason for exclusion: Ineligible study design**

51. Perazzo D, Moore R, Kasparian NA, Rodts M, Horowitz-Kraus T, Crosby L, et al. Chronic pediatric diseases and risk for reading difficulties: a narrative review with recommendations. Pediatr Res. 2022;92(4):966–78. doi:10.1038/s41390-022-01934-y

**Reason for exclusion: Ineligible study design**

52. Pereira MD, Colombatti R, Alvarez F, Bartolucci P, Bento C, Brunetta AL. Sickle cell disease landscape and challenges in the EU:the ERN-EuroBloodNet perspective. Lancet Haematol. 2023;10(8):E687–94. doi:10.1016/S2352-3026(23)00182-5

**Reason for exclusion: Ineligible study design**

53. Pham CD, Hua DT. Clinical guideline highlights for the hospitalist: management of acute and chronic pain in sickle cell disease. J Hosp Med. 2021;16(4):228–9. doi:10.12788/jhm.3556

**Reason for exclusion: No Reproductive health recommendations**

54. Pohl E. Sickle Cell Disease: Considerations for acute pain management in the hematology-oncology ambulatory setting. Clin J Oncol Nurs. 2021;25(5):605–7. doi:10.1188/21.CJON.605-607

**Reason for exclusion: Ineligible study design**

55. Rees CA, Brousseau DC, Ahmad FA, Bennett J, Bhatt S, Bogie A, et al. Adherence to NHLBI guidelines for the emergent management of vaso-occlusive episodes in children with sickle cell disease: A multicenter perspective. Am J Hematol. 2022;97(11):E412-e415. doi:10.1002/ajh.26696

**Reason for exclusion: Ineligible study design**

56. Reeves SL, Peng HK, Wing JJ, Cogan LW, Goel A, Anders D, et al. Changes in hydroxyurea use among youths enrolled in Medicaid with sickle cell anemia after 2014 revision of clinical guidelines. JAMA Netw Open. 2023;6(3):e234584. doi:10.1001/jamanetworkopen.2023.4584

**Reason for exclusion: Ineligible study design**

57. Riley K, Evans MM, Kowalchik K, Adams L, Lucey M. Sickle cell anemia: Best practices for patient-centered care. Nurs Made Incredibly Easy. 2020;18(6):26–33. doi:10.1097/01.NME.0000717640.57159.b5

**Reason for exclusion: Ineligible study design**

58. Rivière E. British Society for Haematology guidelines to improve the care of asplenic patients: Much work done, some remaining and a call for national registries. Br J Haematol. 2024;204(5):1573–6. doi:10.1111/bjh.19379

**Reason for exclusion: Ineligible phenomena of interest**

59. Stanley AY, Wallace JB, Hernandez AM, Spell JL. Anemia in pregnancy: screening and clinical management strategies. MCN Am J Matern Child Nurs. 2022;47(1):25–32. doi:10.1097/NMC.0000000000000787

**Reason for exclusion: Ineligible study design**

60. Trompeter S, Massey E, Robinson S. Position paper on International Collaboration for Transfusion Medicine (ICTM) Guideline “Red blood cell specifications for patients with hemoglobinopathies: a systematic review and guideline.” Br J Haematol. 2020;189(3):424–7. doi:10.1111/bjh.16405

**Reason for exclusion: No Reproductive health recommendations**

61. Walden J, Creary S. Practical guide for disease-modifying medication management of children and adolescents with sickle cell disease. Hematology Am Soc Hematol Educ Program. 2024;2024(1):604–10. doi:10.1182/hematology.2024000587

**Reason for exclusion: Ineligible study design**

62. Wolf J, Blais‐Normandin I, Bathla A, Keshavarz H, Chou ST, Al‐Riyami AZ, et al. Red cell specifications for blood group matching in patients with haemoglobinopathies: An updated systematic review and clinical practice guideline from the International Collaboration for Transfusion Medicine Guidelines. Br J Haematol. 2025;206(1):94–108. doi:10.1111/bjh.19837

**Reason for exclusion: No Reproductive health recommendations**

63. Yui J, Peslak SA, Lambert D, Russell E, Sayani F. Implementation of an evidence-based care pathway for emergency department management of sickle cell vaso-occlusive crisis leads to improved clinical outcomes. Blood. 2019;134:4678. doi:10.1182/blood-2019-123868

**Reason for exclusion: Ineligible study design**
