# Supplementary material for: Evaluating Reproductive Health Recommendations in CPGs for Sickle Cell Disease: An Umbrella Review
Source: Clin Public Health Guidel. 2026 Sep 22;3(4):e70091. doi: 10.1002/gin2.70091 (PMC13618671; doi:10.1002/gin2.70091)
Supplement: Supplementary file 3 [file GIN2-3-e70091-s004.docx]

Additional File 3. Data extraction form

| Who is extracting data | [Enter your name] |
| --- | --- |
| Date of extraction | [Enter date] |
| Key attributes of CPGs |  |
| Title of CPG | [Enter title of CPG] |
| Study Reference | [Enter study reference in APA format] |
| Year | [Enter Year] |
| Type of Publication | ☐ Website |
|  | ☐ Peer-reviewed Publication |
|  | ☐ Institutional internal guideline |
|  | ☐ Other [Specify] |
| Publishing Organization: | ☐ Government |
|  | ☐ Local or state authorities |
|  | ☐ National Professional Organization |
|  | ☐ Institution |
|  | ☐ International Guideline Organization |
| Geographic Location or country | [Enter Location or country] |
| Language of Publication: | [Enter Language] |
| Population Focus | [Enter details about the population focus, such as gender, age group] |
| Target Audience of CPG (select all that apply): | ☐ Healthcare Providers |
|  | ☐ Patients |
|  | ☐ Policymakers |
|  | ☐ Other [Specify] |
| Number of Recommendations in CPG: (Total number of recommendations) | Report total number of recommendations |
| Number of Recommendations in CPG:  (Total number of SCD RP health recommendations) | Check all questions and recommendations. Report only sickle cell RP health recommendations |
| Tool used for process in developing and reporting strength of Recommendation: | ☐ GRADE |
|  | ☐ Oxford |
|  | ☐ SIGN |
|  | ☐ Shekelle |
|  | ☐ Not Mentioned |
| Grading: Approach for Determining RHR Ratings: | ☐ Consensus |
|  | ☐ Evidence-based |
|  | ☐ A Priori Specification |
|  | ☐ Not specified |
| Quality of CPGs using AGREE-II (Please skip this part. We completed it in AgreeTrust) | [Enter DOMAIN scores (not individual items) |
| Scope and Purpose | [Enter Score] |
| Stakeholder Involvement | [Enter Score] |
| Rigor of Development | [Enter Score] |
| Clarity of Presentation | [Enter Score] |
| Applicability | [Enter Score] |
| Editorial Independence | [Enter Score] |
| Overall Guideline Assessment (1-7) | [Enter Score] |
| Additional Comments: |  |
| Key characteristics for *each* Reproductive Health Recommendation (RHR) | RHR #1 (copy as needed for each RHR) |
| Write full name of Reproductive Health Recommendation (RHR) | RHR #1 (copy and paste number and name of SCD RHR here) |
| WHO Domain | ☐ Antenatal, Intrapartum, and Postnatal Care |
|  | ☐ Contraception Counselling and Provision |
|  | ☐ Fertility Care |
|  | ☐ Safe Abortion Care |
|  | ☐ Genetic Counselling |
| Stage of the care continuum addressed by the recommendation (Choose one or more): | ☐ Screening |
|  | ☐ Diagnosis |
|  | ☐ Risk reduction |
|  | ☐ Management |
| Grading: Strength of Recommendation: | ☐ Strong |
|  | ☐ Weak |
|  | ☐ Not specified |
| Grading: Level of Evidence: | ☐ High |
|  | ☐ Moderate |
|  | ☐ Low |
|  | ☐ Very Low |
|  | ☐ Not specified |
| Quality of individual RPRs (AGREE-REX) | [Enter AGREE-REX results for each domain] |
| Clinical Applicability | [Enter Score] |
| Values and Preferences | [Enter Score] |
| Implementability | [Enter Score] |
| Overall Guideline Assessment (1-7) | [Enter Score] |
| Disagreements Resolution: | ☐ Discussion |
|  | ☐ Third Reviewer Involvement |
| Missing Data: | ☐ No missing data |
|  | ☐ Author Contacted |
|  | ☐ Author Non-responsive (Recommendation included with missing data noted) |
| Additional comments: |  |
| Key characteristics for *each* Reproductive Health Recommendation (RHR) | RHR #1 (copy as needed for each RHR) |
| Write full name of Reproductive Health Recommendation (RHR) | RHR #1 (copy and paste number and name of SCD RHR here) |
| WHO Domain | ☐ Antenatal, Intrapartum, and Postnatal Care |
|  | ☐ Contraception Counselling and Provision |
|  | ☐ Fertility Care |
|  | ☐ Safe Abortion Care |
|  | ☐ Genetic Counselling |
| Stage of the care continuum addressed by the recommendation (Choose one or more): | ☐ Screening |
|  | ☐ Diagnosis |
|  | ☐ Risk reduction |
|  | ☐ Management |
| Grading: Strength of Recommendation: | ☐ Strong |
|  | ☐ Weak |
| Grading: Level of Evidence: | ☐ High |
|  | ☐ Moderate |
|  | ☐ Low |
|  | ☐ Very Low |
| Grading: Approach for Determining RHR Ratings: | ☐ Consensus |
|  | ☐ Evidence-based |
|  | ☐ A Priori Specification |
| Quality of individual RPRs (AGREE-REX) | [Enter AGREE-REX results for each domain] |
| Clinical Applicability | [Enter Score] |
| Values and Preferences | [Enter Score] |
| Implementability | [Enter Score] |
| Overall Guideline Assessment (1-7) | [Enter Score] |
| Disagreements Resolution: | ☐ Discussion |
|  | ☐ Third Reviewer Involvement |
| Missing Data: | ☐ No missing data |
|  | ☐ Author Contacted |
|  | ☐ Author Non-responsive (Recommendation included with missing data noted) |
| Additional comments: |  |
